# Non-linear hierarchy of the quorum sensing signalling pathway in bloodstream form African trypanosomes

**DOI:** 10.1371/journal.ppat.1007145

**Published:** 2018-06-25

**Authors:** Lindsay McDonald, Mathieu Cayla, Alasdair Ivens, Binny M. Mony, Paula MacGregor, Eleanor Silvester, Kirsty McWilliam, Keith R. Matthews

**Affiliations:** Institute for Immunology and Infection Research, School of Biological Sciences, University of Edinburgh, Edinburgh, United Kingdom; University of California, Los Angeles, UNITED STATES

## Abstract

*Trypanosoma brucei*, the agents of African trypanosomiasis, undergo density-dependent differentiation in the mammalian bloodstream to prepare for transmission by tsetse flies. This involves the generation of cell-cycle arrested, quiescent, stumpy forms from proliferative slender forms. The signalling pathway responsible for the quorum sensing response has been catalogued using a genome-wide selective screen, providing a compendium of signalling protein kinases phosphatases, RNA binding proteins and hypothetical proteins. However, the ordering of these components is unknown. To piece together these components to provide a description of how stumpy formation arises we have used an extragenic suppression approach. This exploited a combinatorial gene knockout and overexpression strategy to assess whether the loss of developmental competence in null mutants of pathway components could be compensated by ectopic expression of other components. We have created null mutants for three genes in the stumpy induction factor signalling pathway (RBP7, YAK, MEKK1) and evaluated complementation by expression of RBP7, NEK17, PP1-6, or inducible gene silencing of the proposed differentiation inhibitor TbTOR4. This indicated that the signalling pathway is non-linear. Phosphoproteomic analysis focused on one pathway component, a putative MEKK, identified molecules with altered expression and phosphorylation profiles in MEKK1 null mutants, including another component in the pathway, NEK17. Our data provide a first molecular dissection of multiple components in a signal transduction cascade in trypanosomes.

## Author summary

African trypanosome parasites respond to density sensing information in the bloodstream of their mammalian hosts to generate their transmission stage, the stumpy form. Components of this ‘quorum sensing’ signalling cascade are known but their interactions and ordering are not. Here we have dissected the dependency relationships between molecules in the pathway by combinatorial gene knockout and ectopic expression, as well as by detailed phosphoproteomic analysis of one component. Our results provide a first analysis of the signal pathway architecture, revealing that it is non-linear. Moreover, phosphoproteome analysis reveals pathway hierarchy through identifying that the phosphorylation of a NEK kinase component of the pathway is reduced when a predicted upstream kinase is absent. This provides a framework for the coherent dissection of a signal transduction cascade in these parasites that use quorum sensing to control disease spread.

## Introduction

Cells respond to their external environment in order to regulate their proliferation, developmental fate, specialisation or death. This can be in response to environmental cues such as temperature, pH or light, or can be driven by chemical signals generated by other cells of the same species or from competing or co-operating cells occupying the same niche [1]. To respond to such signals, single-celled and multicellular organisms have evolved elaborate signalling pathways in which surface receptors often transduce a signal to protein kinases and protein phosphatases, these transduction cascades eventually driving changes in gene expression either in their nucleus, or by generating phenotypic responses through changes in the abundance or activity of mRNAs or proteins [2–4]. The organisation of these signalling cascades is relatively well conserved in overall structure in eukaryotic organisms, although the individual receptors and transducer kinases and phosphatases are different. In particular, the ability of cells to become quiescent through exiting the cell cycle is a central feature of eukaryotic life, enabling cells to withstand periods of nutrient restriction, or to prepare for cell differentiation [5].

Parasitic protozoa also respond to extracellular signals to regulate their proliferation, tropism and development to ensure their successful transmission to new hosts [6–8]. Among major pathogens such as malaria, many signalling proteins have been identified and individual components have been assigned functions in different cellular processes [9, 10]. However, in most cases the interactions between different components of the signalling pathways are unknown and the connections between regulators of particular processes are not understood, preventing assembly of a coherent regulatory pathway despite high throughput mutant selection and analysis [11]. The ability to assemble these pathways can also be limited by the evolutionary divergence of protozoan pathogens such that the conventional framework established in the crown group eukaryotes may not apply [12, 13]. In consequence, the environmentally regulated control of virulence, transmission competence, tissue tropism or metabolic adaptation is poorly understood in eukaryotic microbial pathogens.

Environmental sensing has particular importance in the life cycle of *Trypanosoma brucei* in the bloodstream of their mammalian hosts. These parasites are responsible for African trypanosomiasis in humans and animals [14, 15] and live extracellularly in the bloodstream, adipose tissue [16] and skin [17] of hosts where they evade immune destruction by a sophisticated antigenic variation process [18]. This exchange of antigen types during chronic infections contributes to the waves of parasitaemia that characterise infection with these parasites [19, 20]. However, a further contributor is environmentally regulated, that being the development of the parasite in the mammalian bloodstream in preparation for its transmission by tsetse flies [21]. Specifically, as trypanosomes proliferate they generate a soluble factor, stumpy induction factor (SIF), that accumulates as parasite numbers increase [22, 23]. At a given density, the SIF signal stimulates the bloodstream parasites to exit their proliferative cell cycle and differentiate to morphologically stumpy forms that are adapted for transmission by tsetse flies [24]. Therefore, this signal-response pathway serves two purposes- it restricts proliferation of the parasite in the host, prolonging host survival, and it also optimises the parasite for its uptake by the disease vector [21].

The soluble signal driving parasite development is not identified despite many years of effort. However, the pathway that transduces the signal is quite well characterised at least in terms of its molecular composition. This is because the action of SIF can be mimicked *in vitro* by cell permeable cAMP analogues which cause cells to arrest but not undergo full development to stumpy forms [23, 25]. This enabled us to carry out a genome-wide RNAi screen for molecules whose silencing renders parasites unresponsive to the SIF mimic signal [26]. Analysis of the genes contributing to resistance identified approximately 30 components and, for several of them, their involvement in physiological stumpy formation was confirmed *in vivo*. Thus, where cells silenced the expression of identified genes they became highly virulent in mice because they did not arrest with the accumulation of SIF. This validated the selectional screen and provided a large compendium of molecules linked to developmental signalling in the parasite. Furthermore, analysis of the molecules identified revealed that they fell into a potential hierarchy with signal processing molecules (specific to cell permeable cAMP) identified, as well as protein kinases and phosphatases and predicted post transcriptional gene expression regulators [24]. Complementing this screen for activators of stumpy formation, a number of inhibitors of stumpy formation have been described [27, 28], including a novel component of the TOR family whose gene silencing drives parasites to become arrested as stumpy like forms, although this was only tested in monomorphic parasites that are naturally incompetent to undergo this developmental transition [29]. Signalling may involve AMPK (also identified in the genome-wide screen for positive regulators of stumpy formation), which becomes phosphorylated during the slender to stumpy differentiation and whose activity regulates quiescence [30].

Despite the identification of molecules linked to stumpy formation, their positioning with respect to one another is unknown. Here we have applied extragenic suppression as a strategy to dissect the quorum sensing signalling pathway in African trypanosomes. This has exploited our ability to create null mutants for pathway components that have lost the ability to respond to the SIF signal and to simultaneously express in the same cells other pathway components under doxycycline regulatable expression both *in vitro* and *in vivo*. This approach has determined the dependency relationships between distinct components of the pathway and the existence of complex non-linear relationships in the pathway. The further analysis of one of the null mutants in the signalling pathway has provided a molecular characterisation of the difference between differentiation competent cells and those lacking a central developmental signalling component.

## Results

### General approach

**Fig 1:**
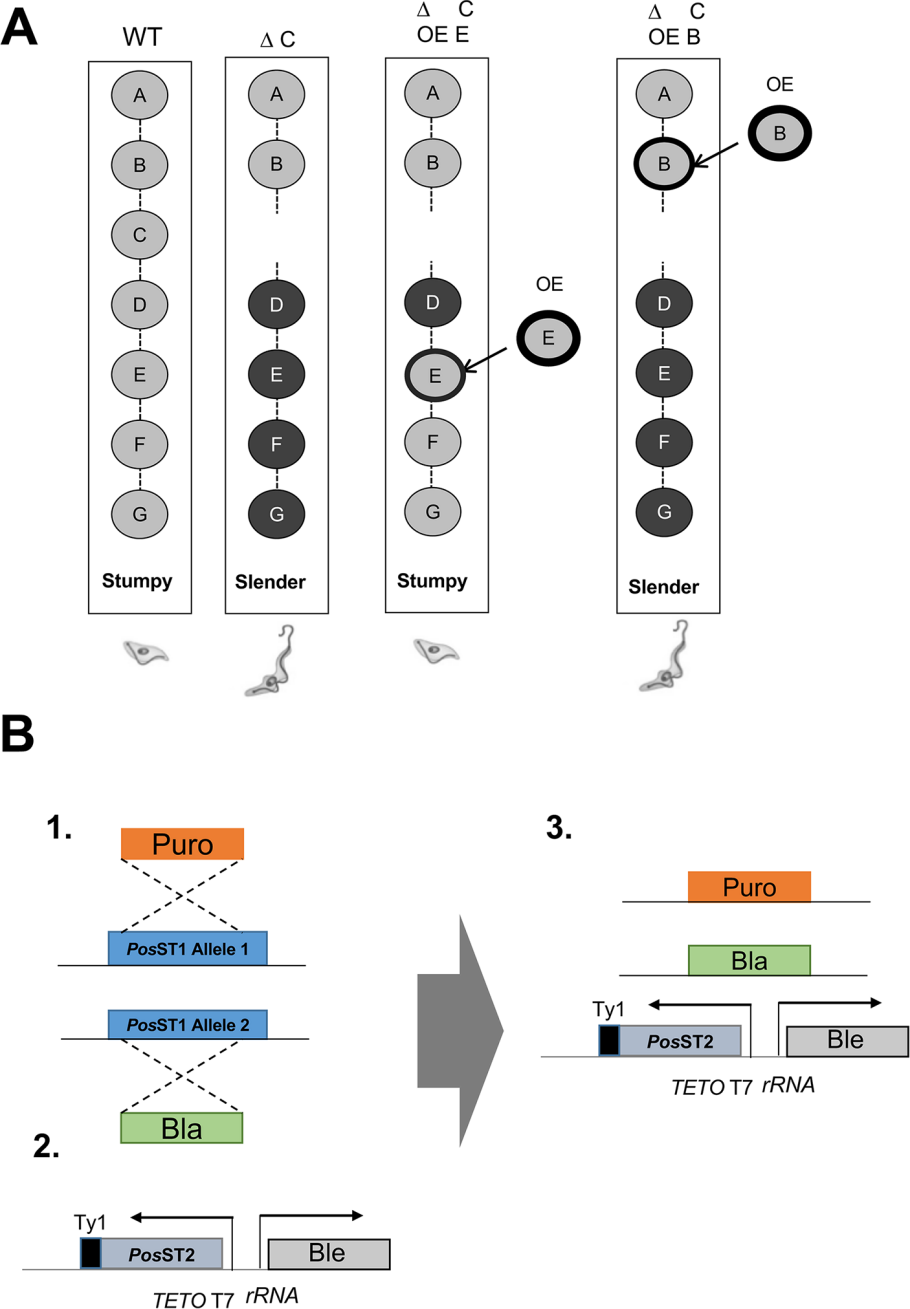
Schematic representation of the experimental approach. **A.** linear signal transduction pathway is represented which, when activated, signals slender forms to differentiate into stumpy forms. If a pathway component is removed (e.g. component C), then stumpy formation is prevented and the parasites remain slender. However, if a component downstream is overexpressed (‘OE’), or a constitutively active mutant expressed, the pathway function can be restored, and stumpy formation occurs. If the overexpressed pathway component is upstream of the breakpoint, the parasites remain slender. **B.** Strategy for the creation of cell lines to test extragenic suppression in the SIF signalling pathway. For creating null mutants, sequential allelic deletion (step 1) used antibiotic resistance cassettes targeting a given PosST (Positive regulator of STumpy formation) gene in the *T*. *brucei* EATRO 1125 AnTat1.1 90:13 parental line, that is capable of tetracycline inducible gene expression. Ectopic expression in the resulting null mutant of a further PosST gene, modified by incorporation of the Ty1 epitope tag sequence was achieved under tetracyclic-inducible regulation (steps 2,3) via the tetracycline/doxycycline regulated T7 promoter (‘TETO T7’).

To investigate the dependency relationships between identified components of the SIF signalling pathway in *Trypanosoma brucei* we used pairwise gene deletion and inducible overexpression to assess the ability of one gene to compensate for the loss of another (Fig 1A). This required the creation of null mutants for the target genes because the kinetics of simultaneous inducible RNA interference and inducible ectopic expression complicate the interpretation of any resulting phenotypes. It also required the use of pleomorphic trypanosome lines since these are competent for developmental progression to stumpy forms *in vivo*, unlike monomorphic lines which are far more easily manipulated but developmentally incompetent.

The creation of null mutants in *T*. *brucei* EATRO 1125 AnTat1.1 90:13 involved a sequential allelic replacement using nested integrative cassettes to replace each target gene copy in the trypanosome’s diploid genome. Thereafter, ectopic overexpression in the null mutant lines was achieved through integration of a construct allowing doxycycline inducible expression *in vivo*, this generating a protein with a TY epitope tag permitting its expression to be monitored (Fig 1B).

Each generated cell line was assessed *in vivo* for their ability to produce stumpy forms, this involving scoring the virulence of the parasites in mice, their accumulation as quiescent G1 arrested cells as parasite numbers increased (indicated by an accumulation of cells with 1 kinetoplast and 1 nucleus), their morphological differentiation to stumpy forms and their expression of the stumpy form specific cell surface marker PAD1[31]. The ability of cells to generate procyclic parasites once harvested from infections and exposed to cis aconitate (CA) was also assessed, this confirming the developmental competence of the parasites: stumpy forms uniformly express the procyclic form surface marker EP procyclin within 2–4 hours of exposure to CA whereas slender forms either do not differentiate, or differentiate asynchronously and inefficiently over 24 hours [32]. In all cases, triplicate infections were carried out for both ‘induced’ and ‘non induced’ infection profiles.

### RBP7 is required for efficient differentiation to stumpy forms

**Fig 2:**
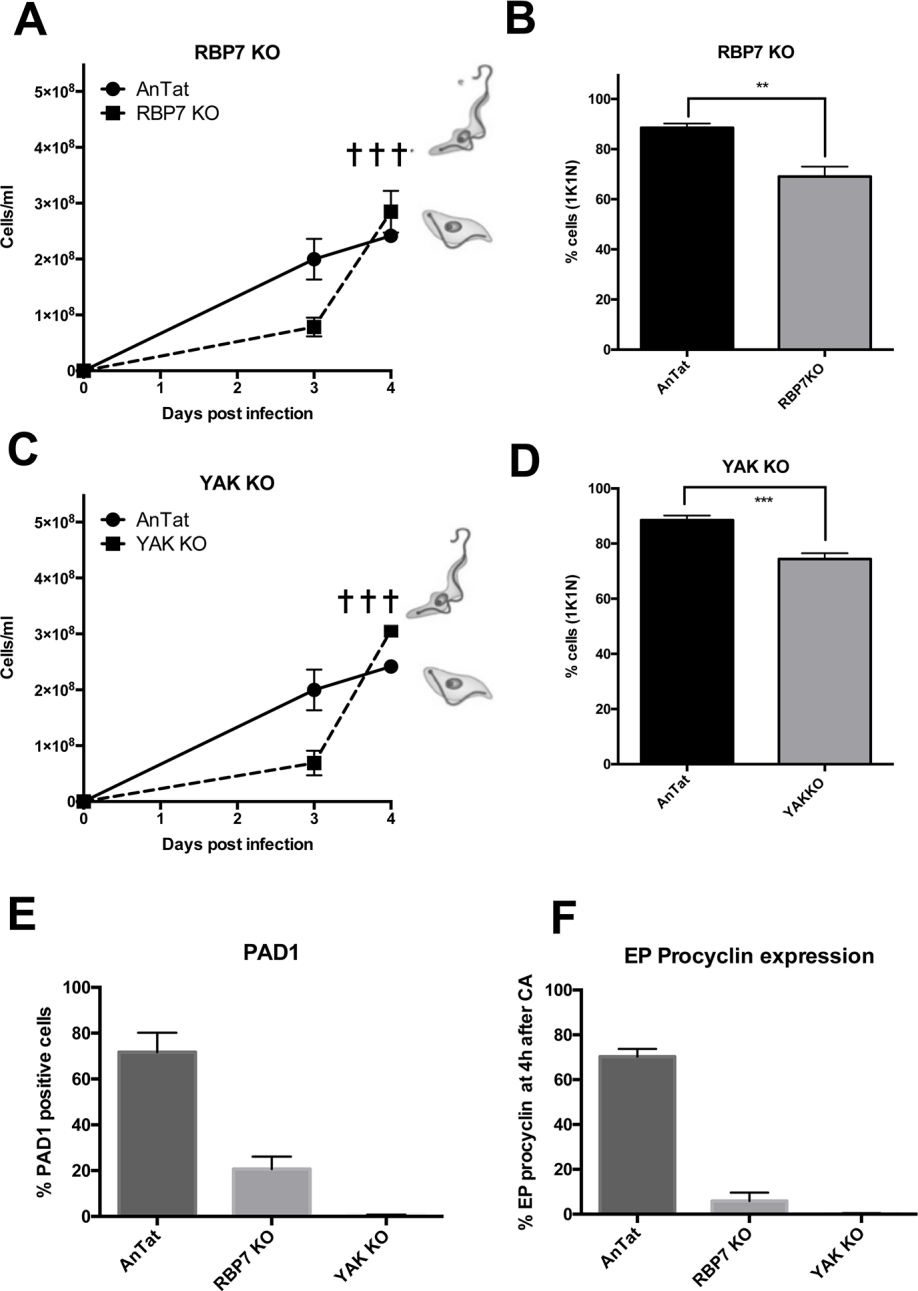
Null mutants for RBP7AB and YAK fail to differentiate to stumpy forms. **A.** Pleomorphic null mutants for *RBP7AB* were assayed for stumpy formation *in vivo*. The parasitaemia of the null mutants (◼) increased without differentiation until the infections were terminated on humane grounds (✝). The parental *T*. *brucei* EATRO 1125 AnTat1.1 90:13 line (●) showed reduced growth from day 3, and by day 4 of infection, were developing to intermediate and stumpy forms, this being represented schematically on the graph. Note that the relative growth of parental and null mutants cannot be directly compared, these being independent cell lines; the parental cells are included to show the progression from slender to stumpy forms in normal infections. **B.** %1K1N of cells on day 4 of infection for the parental (‘AnTat’) or the *RBP7AB* null mutant line. **P<0.005 GLM and Tukey test for multiple comparisons. **C.** Pleomorphic null mutants for *YAK* were assayed for stumpy formation *in vivo*. The parasitaemia of the null mutants (◼) increased without differentiation until the infections were terminated on humane grounds (✝). The parental *T*. *brucei* EATRO 1125 AnTat1.1 90:13 line (●) showed reduced growth from day 3, and by day 4 of infection, were developing to intermediate and stumpy forms, this being represented schematically on the graph. Note that the relative growth of parental and null mutants cannot be directly compared, these being independent cell lines; the parental cells are included to show the progression from slender to stumpy forms in normal infections. **D.** %1K1N of cells on day 4 of infection for the parental (‘AnTat’) or the *YAK* null mutant line. ***P<0.0005 GLM and Tukey test for multiple comparisons. **E.** Cell lines harvested on day 4 of infection, assayed by flow cytometry for PAD1expression. **F.** Cell lines incubated in SDM-79 medium and exposed to 6mM cis aconitate (CA) after which they were assayed for EP Procyclin expression at 4 hours.

Our earlier RNAi analysis had indicated that the predicted RNA binding protein RBP7 (encoded by two tandemly arranged gene copies, *RBP7A* and *RBP7B*) was required for the sensitivity of trypanosomes to cell permeable cAMP and for the SIF-induced differentiation of pleomorphic trypanosomes to stumpy forms *in vivo*[26]. To confirm this, we initially created pleomorphic null mutants for *RBP7AB* using integrative constructs targeting the 5’UTR of *RBP7A* and the 3’UTR of *RBP7B*, the resulting cells being validated by genomic PCR amplification of a fragment of the coding regions using gene specific primers (S1A Fig; other null mutants described in this study are validated in S1B and S1C Fig). Thereafter, the *RBP7AB* null mutants were assessed *in vivo* for their developmental capacity in parallel with wild type *T*. *brucei* EATRO 1125 AnTat1.1 90:13 which are differentiation competent. Fig 2A demonstrates that the null mutant parasites proliferated *in vivo* until infections were terminated on day 4 on humane grounds due to their rapidly ascending parasitaemia. In contrast, the parental control infection showed restricted growth from day 3 and began to develop morphological stumpy forms on day 4. Consistent with this, the control cells showed a significantly higher proportion of parasites with a 1 kinetoplast 1 nucleus configuration of DNA containing organelles[33] reflecting G1 arrest (89%±0.9 vs. 69±2.3% 1K1N in the null mutant on day 4; p = 0.0014, Fig 2B) and, when harvested, expressed the stumpy form marker PAD1 on 72±8.5% of cells compared with 21±5.5% of the RBP7AB null mutant cells (Fig 2E). Furthermore, upon incubation with 6mM CA, 6±3.8% of the RBP7AB null mutant parasites expressed EP procyclin after 4 hours, contrasting with 70±3.4% of the control cells (Fig 2F). All of these parameters confirmed that *RBP7AB* gene deletion reduces the ability of the parasites to arrest as stumpy forms *in vivo*, matching our previous RNAi analysis[26]. However, whilst the inability to completely eliminate stumpy formation seen previously could have been attributable to incomplete gene silencing using RNAi lines, the use of null mutants demonstrated that RBP7AB loss reduced differentiation capacity, but did not completely ablate either stumpy formation or differentiation competence.

### RBP7-promoted growth arrest and development is YAK independent

Having established the effect of *RBP7AB* deletion on stumpy formation, we analysed the interaction between *RBP7* and *YAK* (Tb927.10.15020), encoding a predicted protein kinase of the DYRK family. As with *RBP7AB*, our earlier analysis had demonstrated that *YAK* RNAi reduces stumpy formation [26]and consistent with this, a null mutant for *YAK* resulted in parasites that were virulent *in vivo* (Fig 2C) and showed reduced accumulation in the 1K1N configuration (74±1.2% vs. 89±0.96% in the control, p = 0.0008; Fig 2D). The cells also did not express PAD1 as assessed by flow cytometry (0.7±0.1% vs. 72±8.5%; Fig 2E) and less than 1% of cells expressed EP procyclin 4 hours after exposure to CA (0.4±0.1%; Fig 2F). Hence, contrasting with *RBP7AB* null mutants, where inefficient differentiation was observed, YAK null mutants had very significantly abrogated their ability to differentiate to stumpy forms.

The ability of *RBP7B* ectopic expression to restore differentiation competence to the *RBP7AB* and *YAK* null mutant was then examined. Initially, the consequences for induction of RBP7B ectopic expression were evaluated *in vitro* by growth analysis. S2A and S2B Fig, demonstrate that when RBP7B ectopic expression was induced in either the *RBP7AB* null mutant line, or the *YAK* null mutant line, growth was inhibited there being equivalent RBP7B expression in each mutant (S2C Fig). This matched our prior demonstration that RBP7B ectopic expression in wild type *T*. *brucei* EATRO 1125 AnTat1.1 90:13 cells caused arrested growth *in vitro* and *in vivo*, the latter being accompanied by an accumulation of cells with 1K1N configuration and increased expression of EP procyclin when exposed to 6mM CA [26]. It also indicated that the ectopic expression of Ty-tagged RBP7B alone could restore differentiation in the *RBP7AB* null mutant.

**Fig 3:**
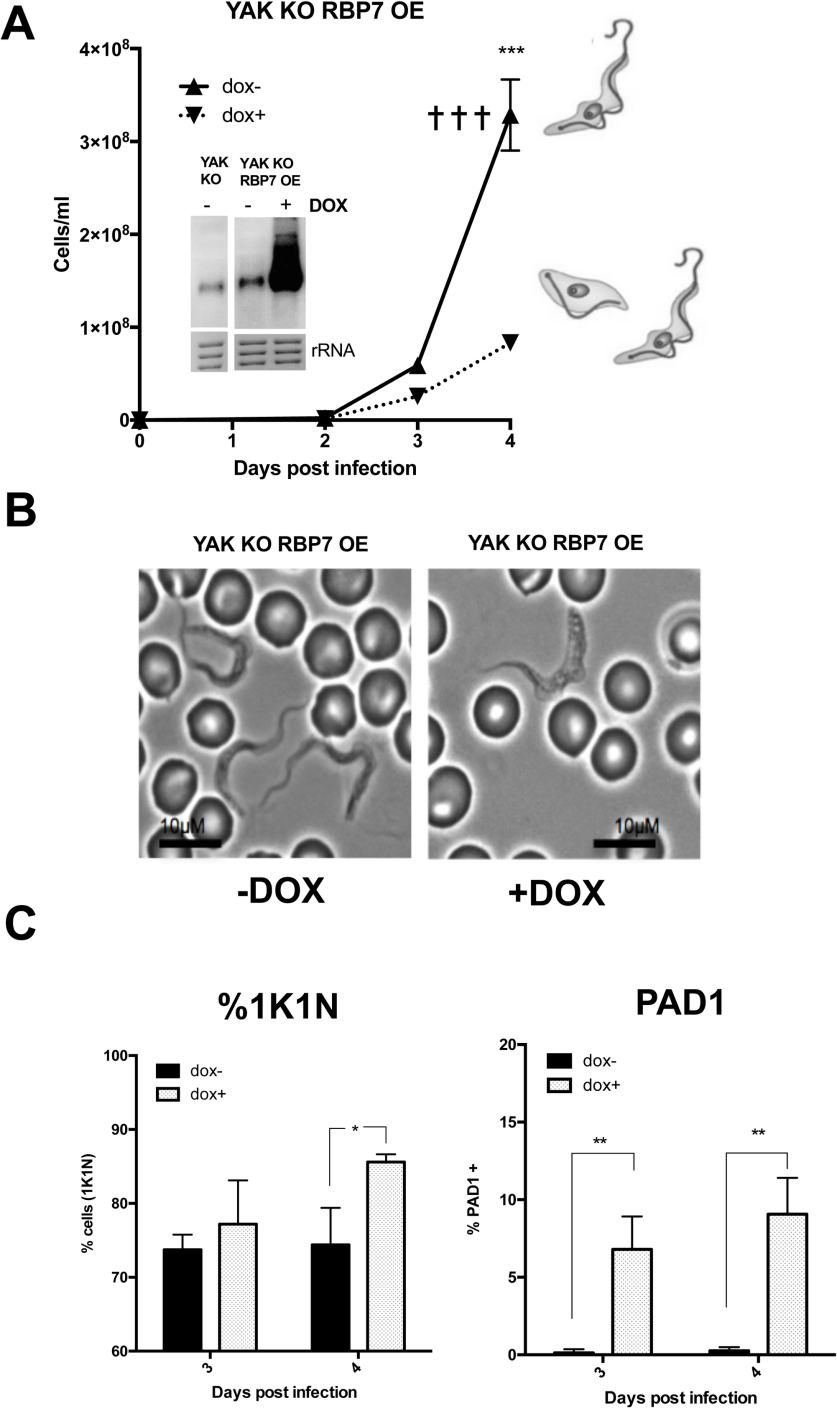
RBP7 ectopic expression induces stumpy formation in YAK null mutants *in vivo*. **A.** Growth of the *YAK* null mutant in which RBP7B ectopic expression was induced (▼), or not (▲) by provision of doxycycline *in vivo*. The inset panel shows a northern blot of *RBP7AB* mRNA expression in the uninduced (-dox) or induced (+dox) parasites on day 4 of infection; an irrelevant lane has been removed. The dominant morphology of the cells at day 4 of infection is indicated schematically. Infection terminated on humane grounds, ✝. ***P<0.0005. **B.** Morphology of *YAK* null mutants induced or not to express RBP7B. Representative cells are shown from day 4 of infection. Cells induced to express RBP7B were more intermediate/stumpy in morphology compared to uninduced cells, which remained slender in morphology. Bar = 10μm. **C.** Left hand panel; cell cycle arrest status of *YAK* null mutants with ectopic RBP7B expression induced or not, *in vivo*, on day 3 and 4 of infection. The proportion of cells with 1K1N was higher where RBP7B expression was induced (* p<0.05). The right-hand panel shows the expression of PAD1 determined by immunofluorescence on day 3 and day 4 of infection. PAD1 expression was detected when RBP7B ectopic expression was induced (** p<0.005).

The consequences of RBP7B ectopic expression in the *YAK* knockout line were then assessed *in vivo* (Fig 3A). Here, the uninduced parasites were highly virulent such that infections were humanely terminated as the parasitaemia ascended beyond 3x10^8^ parasites/ml. In contrast, with RBP7B ectopic expression, the parasites showed slow growth reaching 1x10^8^ parasites/ml on day 4 of infection and a small proportion of cells (~10%) cells with an intermediate/stumpy morphology were observed (Fig 3B). Furthermore, they accumulated with a 1K1N configuration, indicative of cell cycle arrest (86±1% induced vs. 74±5% uninduced on day 4, p = 0.019; Fig 3C left panel). When PAD1 was examined, the capacity of some cells to express this marker was observed (9±1.4 induced vs. 0.3±0.1% uninduced on day 4, p = 0.0029; Fig 3C right panel), this reflecting the proportion of morphologically stumpy forms seen in the population at this relatively early time point. Hence, RBP7B ectopic expression can restore growth arrest and the ability to undergo at least some stumpy transformation in *YAK* KO cells, indicating that RBP7B induced arrest and stumpy formation is not dependent on YAK.

### Protein phosphatase 1 driven stumpy formation is YAK dependent and RBP7 independent

In addition to *RBP7AB* and *YAK*, genome-wide RNAi screens for resistance to the cell permeable cAMP analogue pCPTcAMP identified protein phosphatase 1 as potentially necessary for stumpy formation, this being confirmed *in vivo* by the simultaneous RNAi-mediated silencing of three *PP1* genes (*PP1-4*, *5*, *6*; respectively, Tb927.4.3640, Tb927.4.3630, Tb927.4.3620; >94.90% identity), whereupon stumpy formation was lost[26]. The creation of null mutants for the *PP1-4*, *5*, *6* genes, individually or together, was not successful potentially due to the tandem arrangement of these genes. However, it was possible to explore the ability of PP1-6 ectopic expression to restore stumpy formation in the *RBP7* and *YAK* null mutant lines evaluated above.

**Fig 4:**
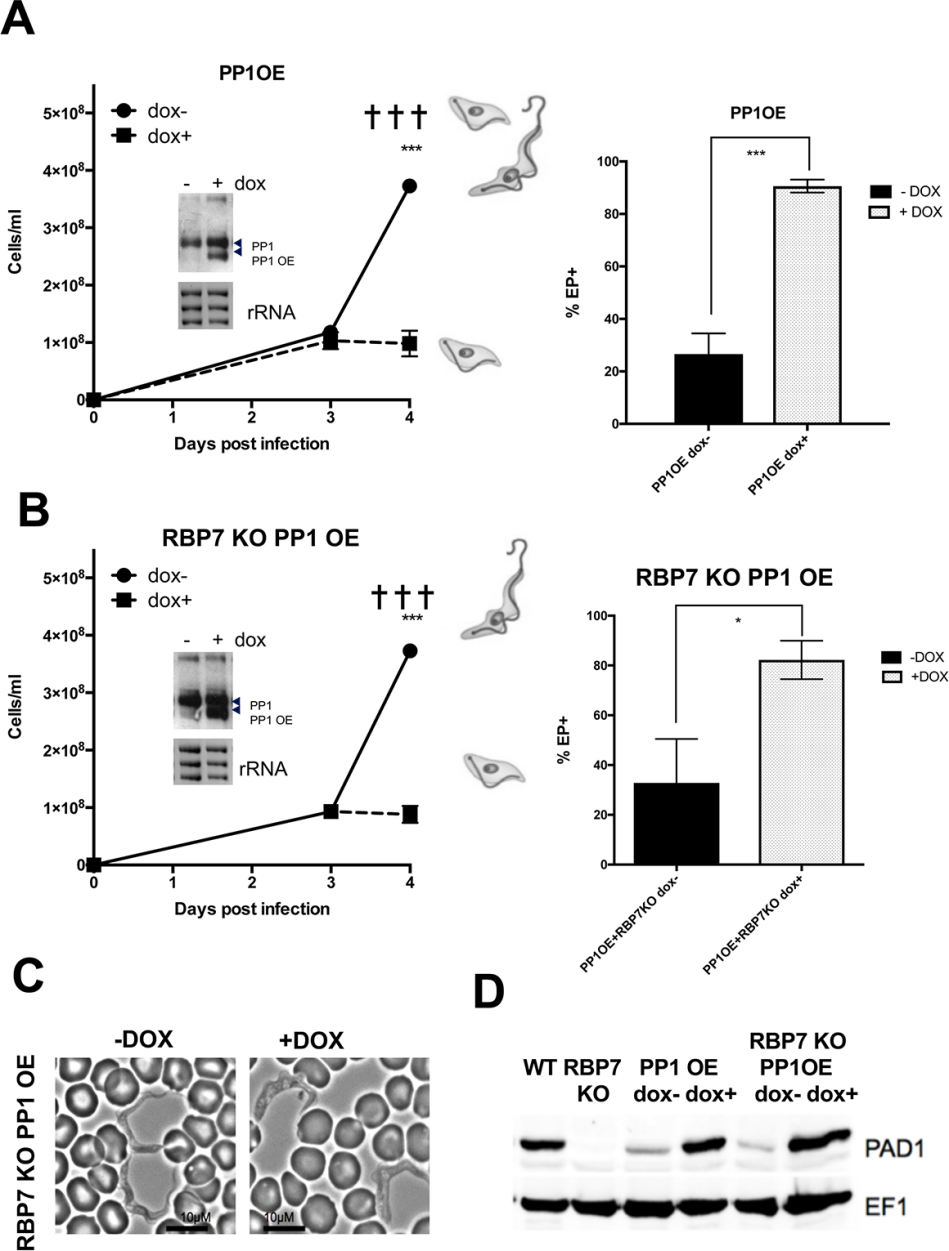
PP1-6 ectopic expression restores stumpy formation in RBP7 null mutants. **A.** Inducible ectopic expression of PP1-6 in *T*. *brucei* EATRO 1125 AnTat1.1 90:13 parental cells (◼, +dox; ●, -dox) (*** p<0.0005). The dominant morphology of the cells on day 4 is shown schematically, which was stumpy in the induced samples and a mixture of slender, intermediate and stumpy in the uninduced samples. The inset northern blot shows PP1 transcript levels in the induced and uninduced cells; rRNA is the loading control. The right panel shows the expression of EP procyclin 4 hours after parasites were harvested on day 4 of infection and incubated in 6mM cis aconitate (*** p<0.0005). Infection terminated on humane grounds, ✝. **B.** Inducible ectopic expression of PP1-6 in *T*. *brucei* EATRO 1125 AnTat1.1 90:13 *RBP7AB* null mutants (◼, +dox; ●, -dox); (** p<0.005). The dominant morphology of the cells on day 4 is shown schematically. The inset northern blot shows PP1 transcript levels in the induced and uninduced cells; rRNA is the loading control. The right panel shows the expression of EP procyclin 4 hours after parasites were harvested on day 4 of infection and incubated in 6mM cis aconitate (* p<0.05). **C.** Morphology of *RBP7AB* null mutants induced (+dox) or not (-dox) to express PP1-6 (RBP7 KO PP1 OE). Bar = 10μm. **D.** Western blot of PAD1 expression in parental parasites (WT), RBP7AB null mutant (RBP7 KO) cells, or in parental cells (‘PP1 OE’) or in the null mutant (RBP7KO PP1 OE) induced (+dox) or not (-dox) to ectopically express PP1-6. The *RBP7AB* null mutant expresses PAD1 upon PP1-6 ectopic expression. EF1alpha provides a loading control.

Initially we investigated the consequences of PP1-6 (Tb927.4.3620) ectopic expression in a wild type *T*. *brucei* EATRO 1125 AnTat1.1 90:13 background *in vitro* (S3A Fig) and *in vivo* (Fig 4A). Here, induction of PP1-6 expression resulted in the rapid cessation of parasite growth (Fig 4A, left) and the appearance of morphologically stumpy forms in rodent infections, despite the low parasitaemia of the induced population. The induced parasites also showed an accumulation of cells with 1K1N configuration (p<0.0001: S4A Fig) and elevated expression of PAD1 (Fig 4D). Their capacity for differentiation to procyclic forms *in vitro* as assessed by EP procyclin expression was also significantly enhanced (p = 0.0002; Fig 4A, right).

Having demonstrated that PP1-6 ectopic expression could precipitate premature differentiation to stumpy forms in a parental background, inducible expression of PP1-6 was tested in the *RBP7AB* and *YAK* null mutant lines. In an *RBP7AB* null mutant background, PP1-6 ectopic expression was accompanied by reduced parasitaemia (Fig 4B) and the appearance of morphologically intermediate-stumpy forms on day 4 of infection (Fig 4C). This was matched by the expression of PAD1 (Fig 4D), the accumulation of 1K1N cells (92±4% vs. 73±1%; p = 0.0095; S4B Fig), and increased capacity for differentiation to procyclic forms when exposed to CA (82±4% vs. 33±10%, p = 0.0114; Fig 4B, right). Hence, ectopic expression of PP1-6 restored differentiation competence to *RBP7AB* null mutants and demonstrated that PP1-6 induced differentiation was RBP7 independent.

**Fig 5:**
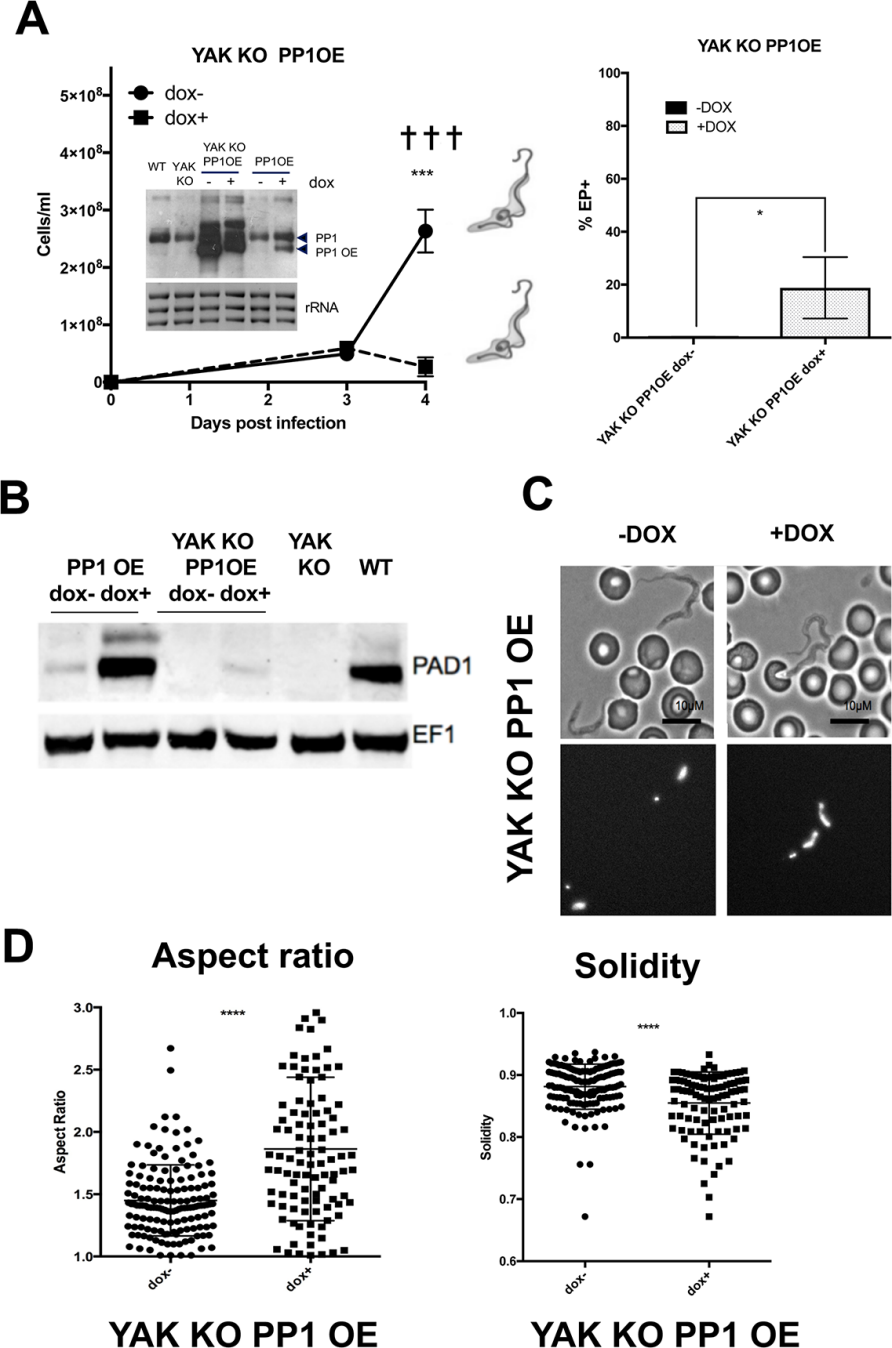
Cytological characteristics of YAK null mutants induced to ectopically express PP1. **A.** Inducible ectopic expression of PP1-6 in *T*. *brucei* EATRO 1125 AnTat1.1 90:13 *YAK* null mutants (◼, +dox; ●, -dox); (*** p<0.0005). The dominant morphology of the cells on day 4 is shown schematically. The inset northern blot shows PP1 transcript levels in the induced and uninduced cells; rRNA is the loading control. The right panel shows the expression of EP procyclin 4 hours after parasites were harvested on day 4 of infection and incubated in 6mM cis aconitate (* p<0.05). **B.** Western blot of PAD1 expression in parental parasites (WT), *YAK* null mutant (*YAK* KO) cells, or in parental cells (‘PP1 OE’) or in the null mutant (YAK KO PP1 OE) induced (+dox) or not (-dox) to ectopically express PP1-6. The *YAK* null mutant does not express PAD1 upon PP1-6 ectopic expression. **C.** Morphology of YAK null mutants induced (+dox) or not (-dox) to express PP1-6. A DAPI stained image of the cells (lower two panels) is also shown to highlight the aberrant nuclear morphology of induced cells with PP1-6 expression. Bar = 10μm. **D.** Analysis of the aspect ratio (left panel) and solidity (right panel) of *YAK* null mutants induced (+dox), or not (-dox) to express PP1-6. (**** p<0.00005).

With a *YAK* null mutant background the picture was more complex. *In vitro*, reduced growth accompanied PP1-6 overexpression (S3B Fig) and *in vivo*, the induction of PP1-6 expression caused the parasitaemia to arrest (Fig 5A) although this was not accompanied by a large accumulation of 1K1N cells (65±2.3% -DOX vs. 73±1% +DOX; p = 0.029;(S4C Fig)) nor effective expression of PAD1 (4±1% -DOX vs.16±3% +DOX, p = 0.015 as determined by immunofluorescence; Fig 5B shows a western blot of PAD1 expression). Furthermore, the capacity of the cells to differentiate to procyclic forms was much less than when PP1-6 was expressed in the parental background (19±6.7% compared to 91±1.4%, p = 0.05; Figs 4A and 5A right panels). Also, when the morphology of parasites induced to ectopically express PP1-6 was examined in the *YAK* null mutant line, stumpy cells were not observed (Fig 5C). Rather, the parasites remained slender but demonstrated aberrant nuclear morphologies, this appearing elongate and often curved or irregular in shape (Fig 5C, lower DAPI stained samples). This morphological phenotype was quantitated using two descriptors- aspect ratio and solidity. The aspect ratio defines the length as a proportion of the width of the organelle, whereas the solidity measures the area of the nucleus divided by its convex area, providing a measure of how concave the nucleus was. An analysis of more than 100 cells (+dox, 102; -dox, 145) demonstrated that the doxycyline treated cells induced to express PP1-6 in the *YAK* background showed a significantly increased aspect ratio and decreased solidity (p<0.00005) (Fig 5D). This nuclear phenotype was not present when PP1-6 was ectopically expressed in the parental cell line, indicating that it was generated only in the absence of *YAK* or with the high level of PP1-6 expression in that line.

Overall this analysis demonstrated that PP1-6 can drive premature differentiation in parental cells and *RBP7AB* null mutants but not in the *YAK* null mutant, where aberrant cells were generated. This indicates that PP1-6 induced stumpy formation is RBP7AB independent and YAK dependent.

### NEK17 driven stumpy formation is RBP7 and YAK dependent

In addition to YAK kinase, a member of the NEK kinase family was also identified in the screen for stumpy inducers, *NEK17* [26, 34]). NEK17 is encoded by a cluster of three almost identical genes (Tb927.10.5950; Tb927.10.5940; Tb927.10.5930; e = 0.0) indistinguishable by RNAi. Therefore, the ability of one representative, Tb927.10.5950 to restore stumpy formation to the *RBP7AB* and *YAK* null mutants analysed above was explored by ectopic expression. *In vitro*, the inducible expression of NEK17 in the parental *T*. *brucei* EATRO 1125 AnTat1.1 90:13 background prevented population growth (S5A Fig). When the same assay was carried out in the *RBP7AB* null mutant, however, the parasites grew as well as uninduced parasites (S5B Fig) despite the effective expression of NEK17, which was at higher level than in the parental background (S5D Fig, left panel). In contrast, when examined in a *YAK* null mutant background, NEK17 ectopic expression resulted in slowed parasite growth (S5C Fig), and expression of the ectopically expressed protein was at the elevated levels seen in the *RBP7AB* null mutant (S5D Fig, right panel).

**Fig 6:**
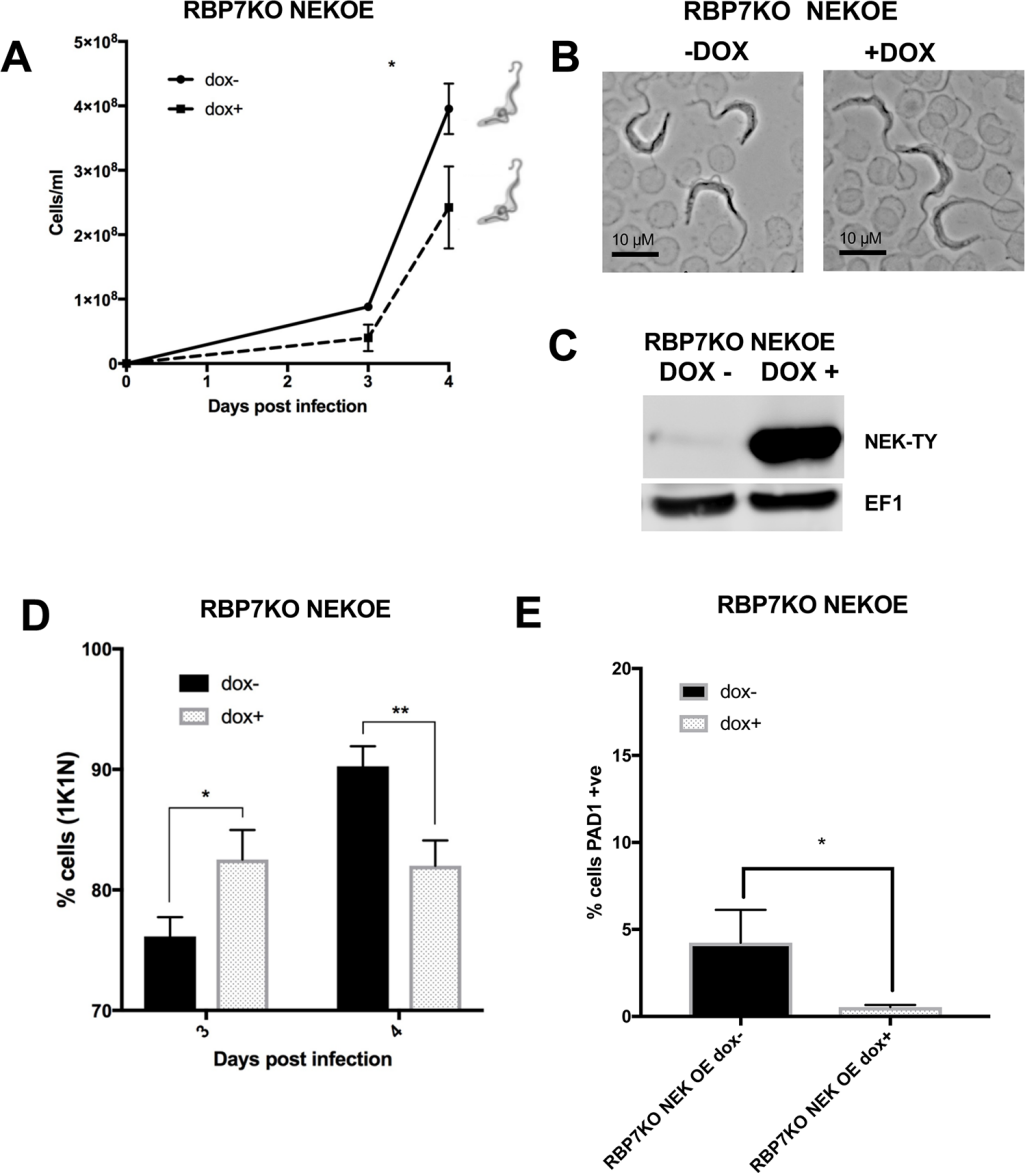
NEK17 induced differentiation is dependent on RBP7. **A.** Growth *in vivo* upon inducible ectopic expression of NEK17 in the *RBP7AB* null mutant. The dominant morphology of the cells on day 4 is shown schematically. Induced (+dox; ◼), uninduced (-dox; ●) over 4 days growth. **B.** Morphology of *RBP7AB* null mutants induced, or not, to ectopically express NEK17. Samples were isolated on day4 of infection. Bar = 10μm. **C.** Western blot demonstrating inducible ectopic expression of NEK17 in the *RBP7AB* null mutant. + dox, induced; -dox, uninduced. Elongation factor 1 alpha (EF1) provides the loading control. **D.** % 1K1N cells upon inducible ectopic expression of NEK17 in the *RBP7AB* null mutant at day 3 and day 4 post infection (* p<0.05; ** p<0.005). **E.** % PAD1 cells upon inducible ectopic expression of NEK17 in the *RBP7AB* null mutant at day 4 post infection (* p<0.05). Less PAD1 expression is detected in the NEK OE due to their lower overall parasitaemia than in the uninduced population (Fig 6A) with respect to RBP7AB KO cells alone (Fig 2E).

**Fig 7:**
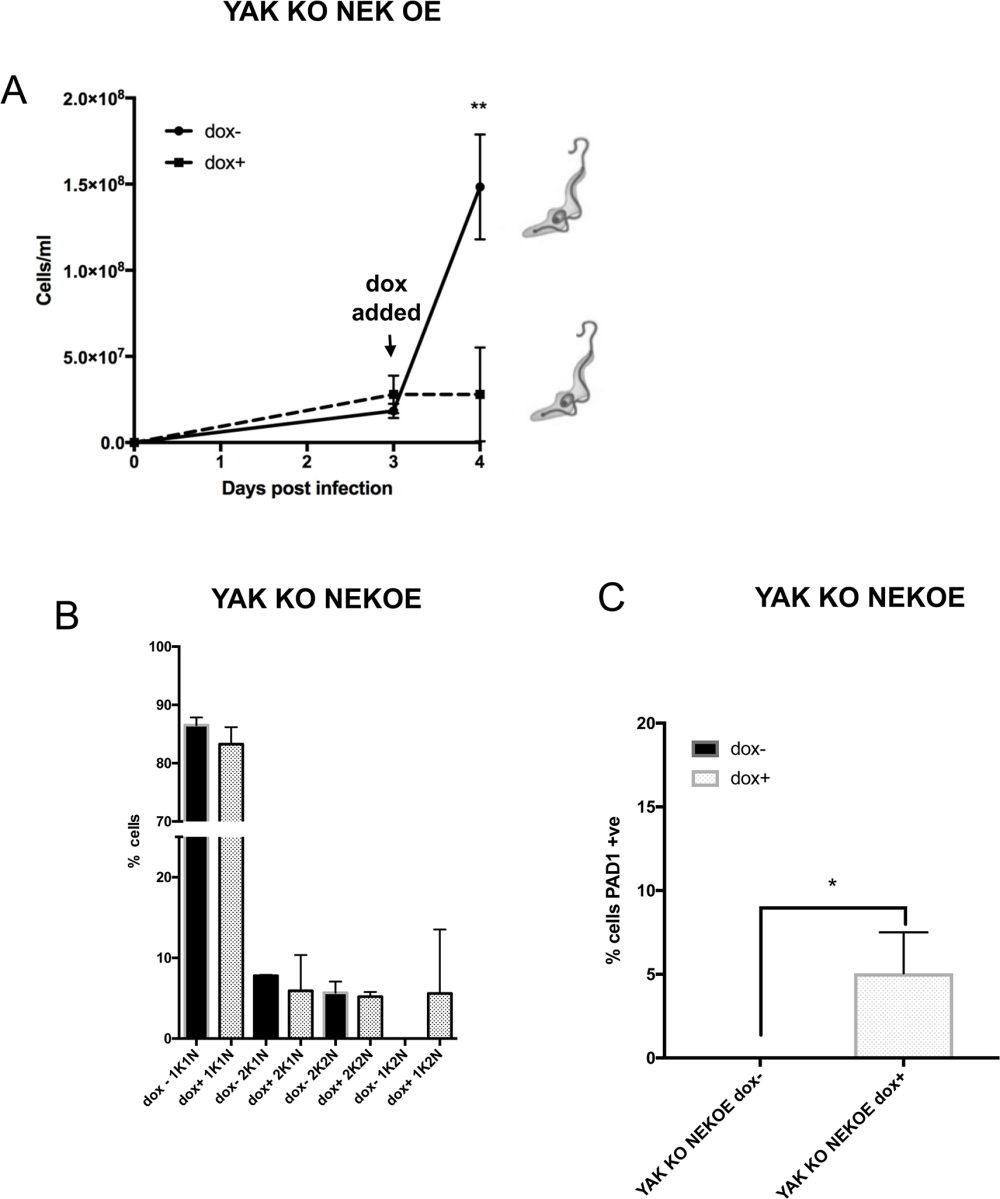
NEK17 induced differentiation is dependent on YAK. **A.** Growth *in vivo* upon inducible ectopic expression of NEK17 in the *YAK* null mutant. Induced (+dox; ◼), uninduced (-dox; ●) over 4 days growth, with doxycycline added on day 3 post infection. A schematic of the dominant morphology of each parasite population is shown. **B.** % 1K1N, 2K1N, 2K2N and the aberrant 1K2N cells upon inducible ectopic expression of NEK17 in the *YAK* null mutant at day 4 post infection. The data represents the mean and standard deviation of 250 cells counted at each time point in each infection. **C.** % PAD1 cells upon inducible ectopic expression of NEK17 in the *YAK* null mutant at day 4 post infection (* p<0.05).

In addition to cell growth *in vitro*, the ability of NEK17 expression to drive physiological stumpy formation was analysed in mouse infections. As in culture, NEK17 overexpression in *RBP7AB* null mutants did not arrest growth *in vivo*; the parasites continued to proliferate, albeit more slowly (Fig 6A), and remained slender in morphology (Fig 6B) despite effective expression of the protein (Fig 6C). The cells also did not accumulate in a 1K1N cell cycle configuration (Fig 6D) nor express PAD1 at an elevated level (Fig 6E). When NEK17 was expressed in a *YAK* KO background *in vivo* cells showed greatly reduced growth (Fig 7A), as seen *in vitro* (S5C Fig). However, this was not linked to G1 arrest; rather there was appearance of the aberrant cytological configuration 1K2N (Fig 7B) and PAD1 expression on some cells albeit at low frequency (~5%) (Fig 7C). Hence, in an *RBP7AB* null mutant background, neither arrest nor morphological change or PAD1 expression was seen, demonstrating arrest mediated by NEK ectopic expression is RBP7AB dependent. In contrast, NEK17 expression arrested growth independently of *YAK* but this caused only very limited development to stumpy forms and cell cycle abnormalities, demonstrating that effective NEK17 induced differentiation is also YAK dependent.

### MEKK1 and TOR4 dependency interactions

So far, our studies had focused on interactions between different positive regulators of stumpy formation. However, inhibitors of stumpy formation have also been identified. Amongst these, *Tb*TOR4 is a ‘slender retainer’[24], whose depletion causes stumpy forms to appear in monomorphic populations which normally cannot undergo this development [29]. To explore whether the same effect was present in physiologically-relevant pleomorphic cells, we generated an inducible RNAi line able to reduce the expression of *Tb*TOR4. This line was then analysed in mice, where induction of *TbTOR4* RNAi generated reduced growth over 6 days (S6A Fig) when the levels of *TbTOR4* mRNA were reduced (S6B Fig). This indicated that, as in monomorphs, *Tb*TOR4 depletion arrested cells prematurely, albeit incompletely, perhaps related to the remaining *Tb*TOR4 after RNAi induction.

The effect of *Tb*TOR4 is expected to be close to the top of a signal transduction pathway if organised similarly to other eukaryotes. Also expected to be close to the top of a conventional signalling pathway are MAP kinase kinase kinases (MEKK) and MAP kinase Kinases (MEK). One such molecule was identified in the genome-wide screen for stumpy inducers, *MEKK1* (Tb927.2.2720) whose *Leishmania major* orthologue LmjF02.0570 is annotated as a Ste11/MEKK (the *T*. *brucei* protein is also named *FlaK* [34] for its flagellar location; www.Tryptag.org). Therefore, we investigated the dependency, if any, between *MEKK1* and *TbTOR4* by creating a *MEKK1* null mutant and then activating *TbTOR4* RNAi in the same cell line. This was anticipated to determine whether the absence of *MEKK1* overrides *TbTOR4* silencing-induced arrest, or not.

**Fig 8:**
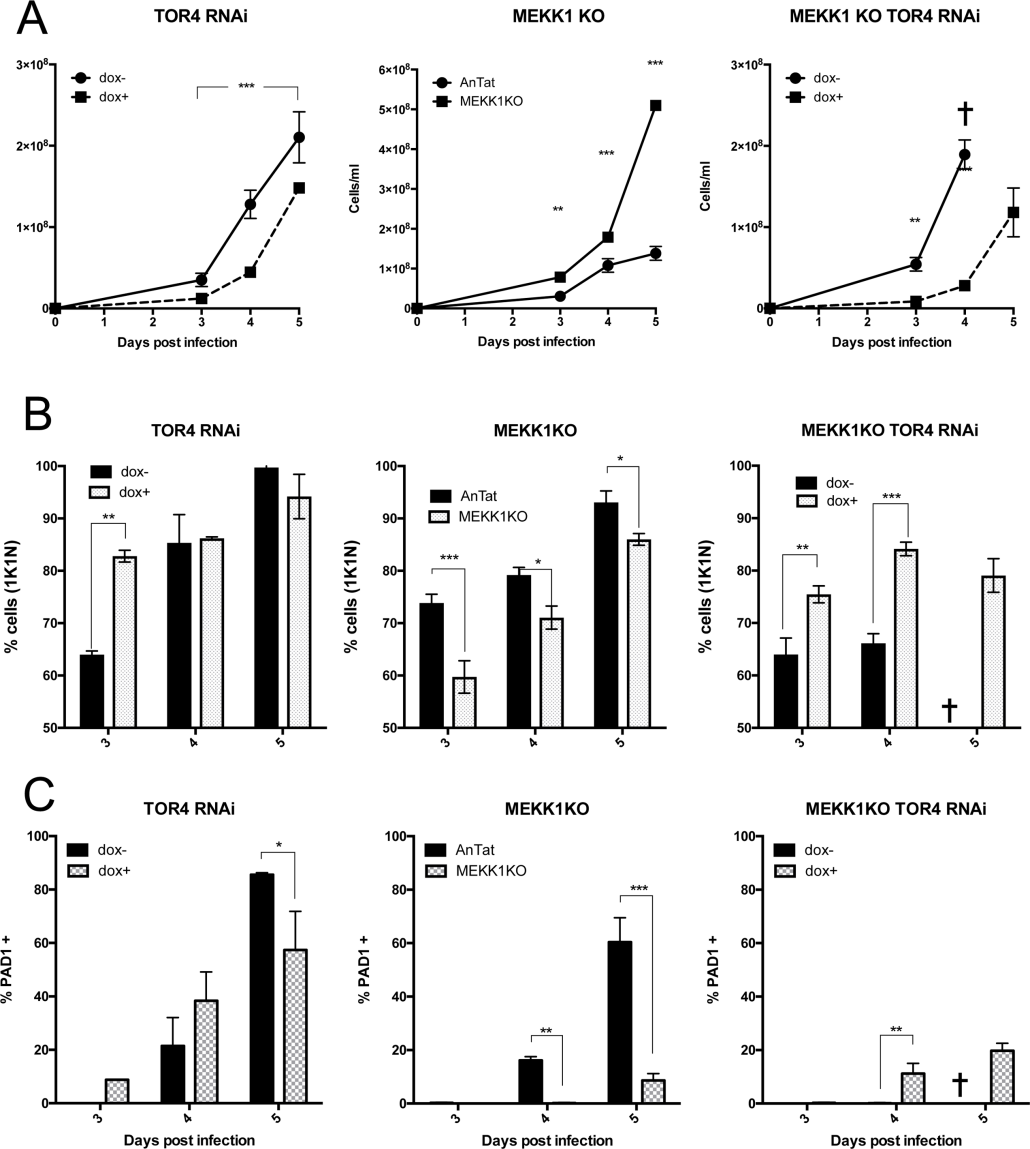
TbTOR4 drives growth arrest independently of MEKK1. **A.** Parasitaemia of the *TbTOR4* RNAi line, *MEKK1* null mutant, or *TbTOR4* RNAi in the MEKK1 null mutant. In the left hand panel, *TbTOR4* silencing promotes stumpy formation; Doxycycline + (RNAi induced; ◼), doxycycline– (RNAi uninduced; ●). In the middle panel, the *MEKK1* null mutants (◼) are more virulent than parental (‘AnTat’) cells (●), consistent with the reduced capacity for stumpy formation. In the right-hand panel, when *TbTOR4* is depleted by inducible RNAi (◼) *MEKK1* null mutants show slow growth compared with *MEKK1* null mutants where *TbTOR4* is not depleted (●). Infections were humanely terminated in the uninduced *MEKK1* KO TbTOR4RNAi infections on day 4 and so day 5 data is absent (✝). *, p<0.05; **, p<0.005; ***, p<0.0005. **B.** Cell cycle progression of the cell lines analysed *in vivo* in Panel A. Left panel, *TbTOR4* RNAi shows enhanced levels of 1K1N cells at day 3 of infection, but by day 4 the uninduced cells have also progressed to stumpy forms. Middle panel, *MEKK1* null mutants show less accumulation in 1K1N than parental (‘AnTat’) cells. Right hand panel; when *TbTOR4* is depleted in the *MEKK1* null mutant, parasites show a higher proportion of 1K1N cells, such that TbTOR4 depletion overrides the absence of *MEKK1*. Infections were humanely terminated in the uninduced *MEKK1* KO *TbTOR4* RNAi infections on day 4 and so day 5 data is absent (✝). *, p<0.05; **, p<0.005; ***, p<0.0005. **C.** PAD1 expression when *TbTOR4* RNAi is induced in parental cells (left hand panel) or in the *MEKK1* null mutant (right hand panel). In the *TbTOR4* RNAi line on day 5 PAD1 expression in the uninduced cells exceeds the induced cells because these cells have progressed to stumpy at high parasitaemia; in contrast, the induced cells show incomplete *TbTOR4* RNAi and cells with effective knockdown express PAD1 early (day 3 and 4), while those without effective knockdown continue to proliferate as slender forms and express less PAD1 because the overall parasitaemia is lower. The middle panel shows less PAD1 is expressed in the absence of *MEKK1* compared with parental (‘AnTat’) cells. Infections were humanely terminated in the uninduced *MEKK1* KO TbTOR4 RNAi infections on day 4 and so day 5 data is absent (✝). *, p<0.05; **, p<0.005; ***, p<0.0005.

As expected, the *MEKK1* null mutant was virulent in mice such that infections had to be terminated by day 5 (Fig 8A, MEKK1, middle panel), this being consistent with their incapacity to generate stumpy forms. However, when RNAi against *TbTOR4* was induced in the *MEKK1* null mutant background, the growth of the null mutant was reduced, indicating that *TbTOR4* silencing overrides the virulence generated by *MEKK1* deletion (Fig 8A; MEKK1 KO TOR4 RNAi). This was supported when the accumulation of cells in a 1K1N configuration was examined (Fig 8B). Thus, *MEKK1* null mutants showed reduced levels of 1K1N cells compared to parental *T*. *brucei* EATRO 1125 AnTat1.1 90:13 cells (Fig 8B centre ‘MEKK1KO’), whereas the induction of *TbTOR4* RNAi in the *MEKK1* null mutant resulted in significantly more 1K1N cells on day 3 and day 4 of infection with respect to when *TbTOR4* RNAi was not induced (Fig 8B right). This demonstrated that *Tb*TOR4 depletion results in the arrest of parasites independently of the presence of MEKK1. Interestingly, however, when the expression of PAD1 was investigated, it was found that *TbTOR4* RNAi in the *MEKK1* null mutant resulted in much less PAD1 expression than when *Tb*TOR4 was depleted in the presence of *MEKK1* (Fig 8C). Thus, on day 4 of infection, *TbTOR4* RNAi in the parental cell line generated 38.4±7.6% PAD1+ve cells, whereas *TbTOR4* RNAi in the *MEKK* null mutant at the same time point, few (11.2±2.2%) cells were PAD1 +ve (Fig 8C). A similar phenomenon was observed on day 5 of infection (*TbTOR4* RNAi induced in parental cells, 57.4±10.2%; *TbTOR4* RNAi induced in the *MEKK1* null mutant, 19.7±1.6%). Apparently therefore, *Tb*TOR4 depletion causes the arrest of cells independently of the presence of *MEKK1*, but *MEKK1* is necessary for the efficient expression of the stumpy marker PAD1. Since arrest precedes the expression of PAD1 protein in the development pathway that results in the formation of stumpy cells, this indicates that both *TbTOR4* and *MEKK1* are involved in cell cycle arrest, but that *MEKK1* is required for efficient development to stumpy forms.

### MEKK1 phosphoproteomic analysis

**Fig 9:**
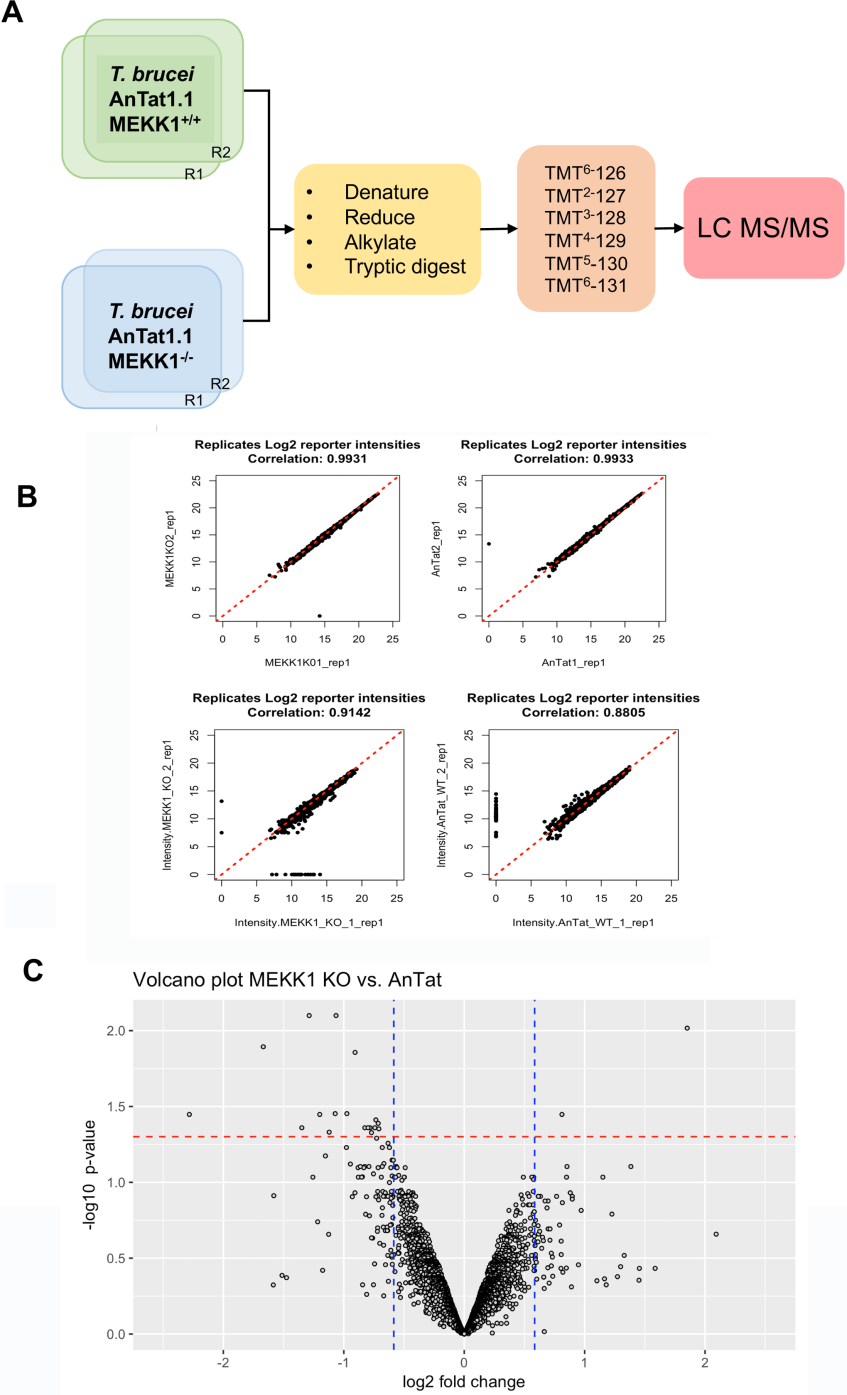
Phosphoproteomic analysis of a MEKK1 null mutant. **A.** Experimental approach. *T*. *brucei* EATRO 1125 AnTat1.1 90:13 and *MEKK1* null mutants were grown in culture and harvested after identical growth profiles and at low cell density to maintain them as the slender developmental form. Samples were isolated, processed, subjected to isobaric mass tagging and then analysed by LC MS/MS. **B.** Reproducibility of the identified protein and phosphoprotein profiles derived from the replicates of the parental (‘AnTat1.1’) and *MEKK1* null mutant cells. **C.** Volcano plot analysis of phosphopeptide changes between the parental and *MEKK1* null mutant. Analyses focused on changes that were statistically significant (adjusted P value <0.05; shown with red dashed lines on the–log10 scale) and at least 1.5-fold different (shown as blue dashed lines on the Log2 scale) between the parental and *MEKK1* null mutant.

Our genetic dissection above attempted to identify dependency relationships between different components of the differentiation control pathway. However, these studies do not inform on molecular interactions between the components, nor distinguish direct or indirect molecular consequences of the perturbations generated. To explore molecular changes associated with a loss of developmental competence in slender forms we analysed the phosphoproteomic changes that accompany deletion of *MEKK1* as this was expected to operate early in the signalling pathway. Hence, parental *T*. *brucei* EATRO 1125 AnTat 1.1 90:13 and the *MEKK1* null mutant cells were cultured in duplicate at equivalent cell density *in vitro* and protein extracted and analysed after isobaric tandem mass tagging (Fig 9A). In total 4767 unique proteins and 2499 unique phosphoproteins were identified; correlations between the replicates were >99% at the peptide level, and at the phosphopeptide level were 0.91 (*T*. *brucei* EATRO 1125 AnTat1.1 90:13) and 0.88 (*MEKK1* null mutant) respectively, demonstrating excellent reproducibility (Fig 9B). The datasets (S1 Table) were then analysed for peptides with >1.5-fold changes in phosphorylation, with an adjusted P value of <0.05, regardless of the direction of change (i.e. more phosphorylated or less phosphorylated in the *MEKK1* null mutant compared to *T*. *brucei* EATRO 1125 AnTat1.1 90:13 cells) (Fig 9C). This identified 19 proteins, of which 17 were 1.5-fold less phosphorylated in the null mutant and 2 were 1.5-fold more phosphorylated. Most interestingly, in the *MEKK1* null mutant we observed significantly reduced phosphorylation of peptides derived from another component of the quorum sensing signalling pathway, NEK 17 (Log2–1.68; adj P = 0.0128). Further, analysis of the phosphosite change revealed that NEK 17 was less phosphorylated in the absence of *MEKK1* at the activation loop threonine 195 (S7 Fig), consistent with reduced kinase activity when cells are less able to generate stumpy forms. Although indirect phosphorylation through other molecular components of the pathway is possible, this invokes the potential for MEKK1 to directly phosphorylate and activate NEK17 in the trypanosome quorum sensing signalling pathway. Other proteins with reduced phosphorylation in the absence of *MEKK1* included the kinetoplastid kinetochore protein KKT4 and RNA regulators ALPH1 and RBP31.

## Discussion

Genome-wide RNAi screens identify the genes whose silencing renders parasites resistant to an imposed selection[35]. This approach was applied to identify genes that confer resistance to cell permeable cAMP, acting as an *in vitro* proxy for SIF-induced differentiation *in vivo*[26]. The resulting screen identified approximately 30 genes, many of which were subsequently validated in individual RNAi lines, and tested for their inability to undergo natural stumpy formation *in vivo*. This confirmed the involvement of many of the hits from the original selection as molecules linked to developmental competence in bloodstream form trypanosomes. However, whilst the list of genes identified suggested the existence of a potential signalling pathway, the ordering and interactions between components of the pathway could not be assumed. Furthermore, it was unclear whether resistance to cell permeable cAMP was enacted through a simple processional linear pathway or whether there was more complexity. One established approach to address this question in conventional genetic systems has been to explore the ability of one molecular mutant to suppress a second mutant and thereby gain understanding of their relative positioning with respect to one another; this also often highlights direct molecular interactions between the respective molecules[36–38]. An alternative approach, epistasis or extragenic suppression, can use the ectopic expression of a wild type or mutant protein to restore a phenotype lost by mutation of another component in a pathway. This also orders the molecules with respect to one another, but does not necessarily imply direct molecular interaction[37]. Here we have used extragenic suppression and phosphoproteomic analysis of a null mutant for a signalling component to dissect the pathway responsible for the generation of stumpy forms in response to the quorum sensing signal, stumpy induction factor, and assigned pairwise dependency relationships between several molecules involved in the process.

The analysis of gene function in trypanosomes has been enormously assisted by the use of inducible gene silencing via RNA interference[39]. However, this has limitations where gene silencing is incomplete, and because the kinetics of gene silencing are such that loss of protein function might not be observed for over 24 hours. These limitations thwarted our early attempts to understand molecular ordering in the stumpy formation pathway, since the ectopic expression of rescue molecules occurred more rapidly than the effective depletion of the partner molecule. Although this issue is now less problematic due to the development of alternative inducible systems based on vanillic acid [40] or cumate [41] control, such that independent gene silencing and ectopic expression can be activated, null mutants provide the best background for complementation studies despite the technical challenge associated with this in pleomorphic cell lines. This was highlighted in our analysis of the differentiation capacity of *RBP7AB* null mutants that retained the capacity for expression of PAD1 in a small proportion of cells, and these were able to differentiate to procyclic forms albeit inefficiently. With the creation of null mutants, low level protein remaining after incomplete gene silencing by RNAi can be eliminated as an explanation for this inefficient differentiation and it is clear that loss of these molecules does not completely eliminate differentiation capacity. The importance of the use of null mutants for the interpretation of pathway dependencies has been well recognised in the genetic dissection of cell lineage determination in *C*. *elegans*[37].

**Fig 10:**
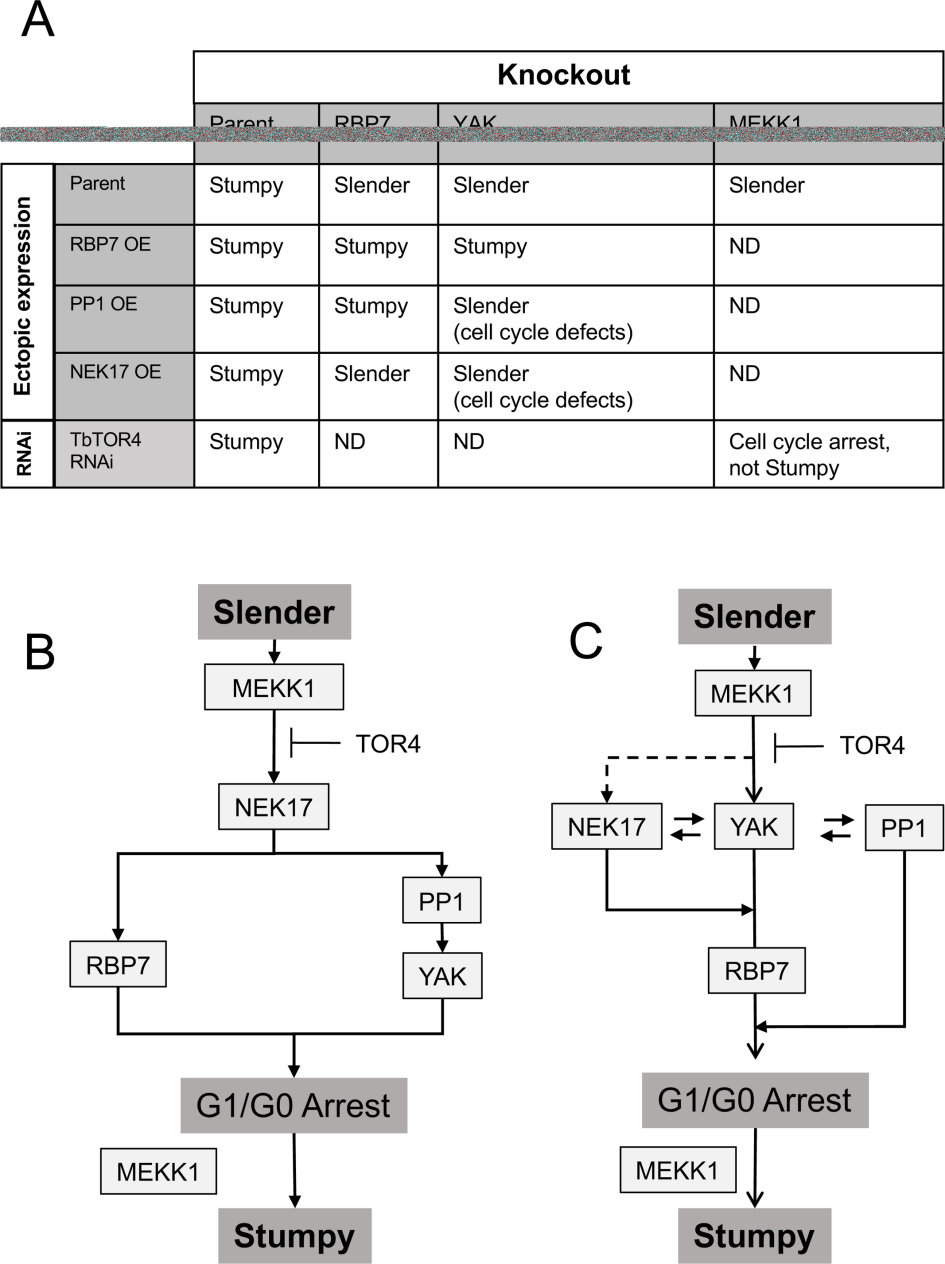
Pathway models and dependency relationships for the interactions studied in this work. **A.** A summary of the ectopic expression and gene knockout or RNAi combinations used in this study. **B.** In this model, the pathway is branched but neither branch is redundant. **C.** In this model, the pathway is unbranched but the presence of *YAK* is necessary for PP1-6 to generate stumpy forms when expressed. RBP7 is required for the NEK17 expression induced growth phenotype; in *YAK* null mutants NEK17 expression generates slow growth. MEKK1 is needed for cell cycle arrest, although this can be overcome when TbTOR4 is depleted. However, MEKK1 is needed for the effective development of stumpy forms in TbTOR4 arrested cells. The dotted line indicates the potential for MEKK1 to directly or indirectly phosphorylate NEK17 as determined by phosphoproteomic analysis.

Analysis of the combination of gene knock out and ectopic expression for several components identified from our RNAi screen highlighted potential features of the control pathway (Fig 10A). Thus, both *RBP7AB* and *YAK* knockout lines prevented efficient development to stumpy forms, and this was restored when RBP7B was ectopically expressed in either line. This confirms the involvement of both genes in the stumpy formation *in vivo* and potentially places RBP7B downstream of YAK, an order that might be anticipated given the predicted role of RBP7B as a gene expression regulator[24, 42]. However, when PP1-6 was ectopically expressed in the same null mutant lines, stumpy formation was observed for the *RBP7AB* null mutant indicating that PP1-6 can promote stumpy formation independently of the presence of RBP7AB. For YAK, the picture was more complex. Although growth inhibition was observed *in vitro*, analysis of PPI-6 expression in the *YAK* KO line *in vivo* highlighted that growth retardation was not accompanied by the hallmarks of stumpy formation: the cells did not activate the expression of PAD1, nor were they capable of efficient differentiation to procyclic forms. Instead, the parasites exhibited a slender morphology and cytological abnormalities, with the nucleus becoming elongate and misshapen. Interestingly, this phenotype was not seen when PP1-6 was expressed in a parental background or the *RBP7AB* null mutant but was restricted to the *YAK* null mutant. The trypanosome genome contains 7 *PP1* genes, of which a cluster of 3 genes, indistinguishable by RNAi, were identified in the screen for regulators of stumpy formation (*PP1-4*, *PP1-5*, *PP1-6*)[26]. The predicted encoded proteins share 95–99% identity. RNAi silencing of all *PP1* genes in procyclic forms generates slow growth (40% of control growth) and an accumulation of mitotic and post mitotic cells[43], whereas specifically depleting *PP1-1* to *PP1-3* in procyclic forms generates altered positioning of the nucleus and kinetoplast[44]. Okadaic acid treatment (inhibiting PP1 and PP2 activities *in vitro*) also caused an accumulation of multinucleate cells[45]. Hence, the phenotype observed with PP1-6 expression in the *YAK* null mutant background is similar to organellar defects seen with other perturbations of *PP1* genes, and may represent activity of PP1-6 against inappropriate targets in the absence of YAK.

Contrasting with PP1-6, the slow growth and differentiation phenotype induced by NEK17 ectopic expression was RBP7AB dependent, being absent in the *RBP7AB* null mutant. NEK17 is a member of the expanded NEK family in *T*. *brucei* which includes NEK12.2 (RDK2), that has been proposed to act as a negative regulator of differentiation to procyclic forms such that its depletion precipitates procyclic formation[34]. When ectopically expressed in a *YAK* null mutant, NEK17 inhibited the growth of parasites but only a few stumpy cells were generated. This indicates NEK17 induced stumpy formation is *RBP7AB*-dependent and that, as with PP1-6, its normal function may also be YAK-dependent. Interestingly, the expression level of NEK17 that was detectable in the parental and *YAK* or *RBP7AB* null mutants differed. Thus, parental cells, with an intact stumpy formation pathway consistently expressed less NEK17 than either of the null mutant lines. Although individual differences between distinct cell lines cannot be eliminated, NEK17 may be subject to regulatory feedback such that its expression is restricted in developmentally-competent cells.

As well as promoters of the differentiation step, inhibitors of the differentiation from slender to stumpy forms have been identified. In particular, a component of a novel TORC complex, *Tb*TOR4, has been identified that prevents the differentiation of monomorphic cells to stumpy forms[29]. To evaluate this finding in a physiologically relevant context, we initially explored the consequences of *Tb*TOR4 inducible depletion in pleomorphic bloodstream forms and confirmed that they too exhibited slow growth and premature G1 arrest upon *Tb*TOR4 loss, although this was incomplete probably due to incomplete transcript depletion. Thereafter, we explored the interaction between *Tb*TOR4 and the predicted MAP Kinase Kinase Kinase (MEKK1) since this too would be predicted to operate early in a signalling cascade if structured in a similar way to conventional eukaryotic pathways. As expected, the *MEKK1* null mutant was virulent *in vivo* and did not generate stumpy forms. However, when *Tb*TOR4 was depleted in the *MEKK* null mutant, slowed growth and G1 arrest was observed similar to the depletion of *Tb*TOR4 alone. This demonstrates that the growth effects of *Tb*TOR4 depletion act independently of MEKK1. However developmental competence after *Tb*TOR4 depletion was not complete in the absence of MEKK1; instead, the cells expressed low levels of PAD1. This may reflect parasites entering cellular quiescence reversibly before committing to irreversible stumpy formation and full PAD1 expression. This would be consistent with our previous mathematical modelling of the transition from proliferative slender forms where cells undergo commitment to differentiation only after cell cycle arrest[46]. It is also consistent with PAD1 being a marker for later stages in the differentiation programme, its mRNA being expressed prior to the expression of PAD1 protein that accompanies morphological transformation[21, 46]. This highlights that *Tb*TOR4 may be needed to prevent reversible cell cycle arrest, similar to the signal-induced reversible quiescence in several eukaryotic systems[47–51]. This is also compatible with the effects of AMPK on trypanosome cellular quiescence observed previously [30]. In contrast, MEKK1 is needed for both effective cell cycle arrest and stumpy formation.

The molecular consequences of depletion of MEKK1 were also evaluated by phosphoproteomic analysis of isobaric mass tag labelled samples. In this analysis, it was important to use pleomorphic slender cells grown in culture so that the mutant and parental (MEKK1+) population could be assessed at an equivalent cell density and growth phase, maximising the ability to detect phosphorylation differences between cells with or without MEKK1. As a protein kinase, direct substrates of MEKK1 might be detected in the null mutant line as molecules demonstrating reduced phosphorylation, although phosphorylation changes in either direction could also result indirectly from the absence of MEKK1. Interestingly, we found that another SIF pathway component, NEK17, showed less phosphorylation in the MEKK1 null mutant line. Furthermore, analysis of the phosphorylation site indicated that it was positioned at the activation loop threonine 195 of the NEK kinase and therefore potentially able to regulate its activity. This provides support for NEK17 being positioned downstream of MEKK1 in the differentiation control pathway although direct interaction remains to be demonstrated. Also identified were several hypothetical proteins, and also proteins linked to gene expression (ALPH1, an mRNA decapping enzyme[52], and the post transcriptional repressor RBP31[53]) and cytoskeletal or protein trafficking control. Regulation of development through the action of RNA binding proteins would be expected given the emphasis of post transcriptional control of the trypanosome genome, such that differential phosphorylation of these proteins may affect their ability to bind RNA or interact with other regulatory or RNA binding proteins to retain cells in a slender form, or permit differentiation to stumpy forms. All of these molecules change phosphorylation status in the absence of MEKK1 but their involvement as slender retainers or stumpy inducers awaits individual analysis.

In Fig 10A we summarise the data generated in our study and in Fig 10B and 10C present alternative (non-exhaustive) models for the combinatorial interactions explored. In one interpretation (Fig 10B), there are two branches leading to G1 arrest and then stumpy formation, one dependent upon YAK, the second dependent on RBP7. Thus, PP1-6 can drive stumpy formation in the absence of *RBP7AB*, but not in the absence of *YAK*, where cell cycle defects are generated. In contrast, NEK17—potentially a direct substrate of MEKK1—is dependent upon both RBP7 and YAK, such that RBP7 is needed to generate arrest and stumpy formation, whereas the absence of YAK generates cell cycle defects and the cells remain slender. Although bifurcated, each branch is not redundant, however, because the loss of individual pathway components on either branch (e.g. YAK or RBP7AB) prevents efficient stumpy formation. Perhaps, therefore, the amount of signal through each branch determines the differentiation response. Notably, the ability of *RBP7AB* null mutants to differentiate at very low level may be contributed by SIF-independent differentiation, a mechanism proposed to operate with disruption of VSG expression site activity[54]. An alternative pathway structure (Fig 10C) is unbranched, with NEK17 and PP1-6 acting downstream of YAK but with each dependent on the presence of YAK for their normal activity; in the absence of YAK, cell cycle defects and growth inhibition arise when either NEK17 or PP1-6 are ectopically expressed. Inevitably a complication in the interpretation of these interactions is that the molecules may have other functions in the cell that are disrupted upon their expression or deletion, exemplified by the inability to knock down the expression of PP1-4, 5, 6 without inducing a strong growth phenotype in bloodstream form cells[26].

In conclusion, our work has highlighted (i) the potential for the differentiation signalling pathway to be non-linear, and (ii) that cell cycle arrest does not inevitably lead to stumpy formation, as exemplified by the *Tb*TOR4-induced arrest, that does not generate PAD1+ cells unless there is MEKK1 present. It has also demonstrated that to accurately interpret the phenotypes resulting from perturbation in molecules proposed to be involved in differentiation requires pleomorphic cell lines and detailed monitoring of physiological stumpy formation through several cytological parameters (G1 arrest, PAD1 expression, morphology and differentiation capacity or procyclic formation). When combined with extragenic suppression and phosphoproteomic analysis in a null mutant background, the systematic piecing together of the components of the signalling pathway becomes possible, as do the wider molecular events linked to remaining as slender forms, or committing to development.

## Materials and methods

### Ethics statement

All animal experiments were carried out after local ethical approval at the University of Edinburgh Animal Welfare and Ethical Review Body (approval number PL02-12) and were approved under the United Kingdom Government Home office licence P262AE604 to satisfy requirements of the United Kingdom Animals (Scientific Procedures) Act 1986. Infections were carried out in adult MF1 mice.

### Trypanosomes

Experiments were carried out using the *Trypanosoma brucei brucei* EATRO 1125 AnTat1.1. 90:13 line[55]. *In vitro*, parasites were cultured in HMI-9[56]. Transfection of pleomorphic AnTat 90:13 strains was performed using either cells grown *in vitro* or harvested from mouse blood[57]. In the latter case, whole blood was harvested from a mouse showing slender parasitaemia and added to 25 ml HMI-9 + 10% FCS and allowed to settle for 4–6 hours. 20 ml of this culture was then carefully pipetted from the top and transferred to a new flask containing another 20 ml media. Any remaining blood was allowed to settle overnight. The following day media was pipetted from the top, transferred to a new flask and used for transfection. 2-3x10^7^ parasites at a density of 6x10^5^-1.2x10^6^/ml were centrifuged for 5 minutes at 1000g and washed in TDB (Trypanosome Dilution Buffer; Trypanosome Dilution Buffer: 5 mM KCl, 80 mM NaCl, 1 mM MgSO_4_, 20 mM Na_2_HPO_4_, 2 mM NaH_2_PO_4,_ 20 mM glucose, pH7.4). They were resuspended in 50–100 μl Amaxa buffer (Lonza) mixed with the DNA. The sample was then transferred to a cuvette and electroporated in an Amaxa Nucleofector II using program Z-001 –Free Program Choice. The cells were immediately transferred to a flask containing 30ml pre-warmed media and serially diluted 1:10 and 1:100 in 2 additional flasks. After incubation at 37°C for minimum 6 hours, antibiotic was added to each flask for selection of transfectants. Antibiotic concentrations used for parasite transfection were: Hygromycin (0.5μg/ml), puromycin (0.5μg/ml), phleomycin (1.5μg/ml), G418 (2.5μg/ml), Blasticidin (10μg/ml). The cultures were plated in 1ml volumes in 24-well plates. Positive transfectants were identified microscopically and diluted into antibiotic-containing media 4–8 days post transfection. For inducible expression and/or RNAi induction, Tetracycline was used at 1μg/ml.

### Construct generation

Constructs were generated using standard molecular biology protocols or by Gibson assembly. For the latter, the assembly reaction comprised 50–100 ng of vector with 2–3 fold excess of each insert in a 10 μl volume. 10 μl of 2x Gibson Assembly Master Mix was then added and the reaction incubated at 50°C for 15 minutes.

Null mutants were created by replacing the YFP and TY tags of the pEnT6B-Y and pEnT6P-Y vectors[58] with fragments of the 3’ and 5’UTRs of each target gene. Restriction digestion of these plasmids between the 3’UTR and 5’UTR sequences produced 2 linear constructs, containing resistance markers to blasticidin and puromycin respectively, which were capable of homologous recombination with, and replacement of, target gene alleles. The pEnT6B-Y construct contained external UTR fragments located distal to the ORF relative to the internal UTR fragments in the pEnT6P-Y construct. This meant that when the pEnT6B-Y construct was transfected into the AnTat 1.1 90:13 strain in the first of 2 sequential transfections, one allele of the target gene including the internal UTR fragments was deleted. When the second pEnT6P-Y construct was transfected, recombination occurs only with the remaining target gene allele, producing a double knockout (dKO) line resistant to both antibiotics. Ectopic expression was achieved using as a construct backbone pDEX577Y[58], that integrates in to the 177bp repeat minichromosome region. Drug selection was mediated by a bleomycin resistance cassette transcribed by a constitutive rRNA promoter; inducible expression was achieved using a T7 promoter with a tetracycline operator sequence.

### Procyclic differentiation assay

Parasites purified from whole blood were resuspended at 3x10^6^/ml in SDM-79 +10% FCS media supplemented with 6mM *cis*-aconitate (Sigma) to induce procyclic differentiation and incubated at 27°C/5% CO_2_. After 0, 4 and 24 hours, 1 ml culture was removed, washed and fixed in 2% formaldehyde/0.05% gluteraldehyde for EP procyclin expression analysis by flow cytometry.

### Flow cytometry

Samples were prepared in 5ml FACS tubes (BD Biosciences). Cells were fixed in 2% formaldehyde/0.05% gluteraldehyde at 4°C overnight. Samples were centrifuged at 2000g for 7 minutes, washed in PBS and blocked in 200 μl 2% BSA/PBS for 40 minutes or overnight. They were centrifuged again and resuspended in primary antibody in 2% BSA/PBS for 1 hour or overnight at 4°C. Cells were then washed again and resuspended in fluorescently conjugated secondary antibody for 40 minutes at 4°C. The cells were washed again and finally resuspended in 500 μl 0.2 μg/ml 4’,6-diamidino- 2-phenylindole (DAPI)/PBS. Antibody concentrations are listed below.

### Immunofluorescence assay

At least 2x10^5^ cells were centrifuged at 1000g for 5 minutes, washed in ice-cold PBS and resuspended in 125 μl ice-cold PBS. 75 μl 8% paraformaldehyde in PBS was added and the tube mixed by inverting. The sample was incubated on ice for 10 minutes. 500 μl PBS was added and the tube centrifuged at 1000g for 5 minutes. The cells were resuspended in 0.1 M glycine/PBS and stored at 4°C.

Samples were resuspended in 15 μl PBS and pipetted onto slide wells pre-treated with 15 μl 0.1 mg/ml poly-L-lysine (Biochrom). Slides were incubated in a humid chamber for 1 hour to allow the cells to adhere. For intracellular proteins, 0.05% Triton X-100 was added for 5 minutes. The wells were washed 3 times in PBS and then blocked in 20% FCS/PBS for 45 minutes. Excess liquid was removed and primary antibody in 20% FCS/PBS was applied for 1 hour. The wells were washed 5 times and then fluorescent dye conjugated secondary antibody in 20% FCS/PBS added for 1 hour. Secondary antibody was aspirated and 10 μg/ml DAPI applied for 1 minute. The wells were washed 5 times in PBS. The slides were removed from the humid chamber and dried for 10 minutes at 37°C, then mounted and dried. Antibody concentrations used are shown in Table 1.

**Table 1:** Antibodies used in various applications in this study.

rabbit α-PAD1 (Eurogentec)	Western	1:1000
rabbit α-PAD1 (Eurogentec)	IFA	1:1000
rabbit α-PAD1 (Eurogentec)	FACS	1:200
mouse α-EF1 (Millipore)	Western	1:7000
mouse α-EP procyclin (VH Bio)	FACS	1:500
mouse α-BB2 (in house)	Western	1:20
mouse α-BB2 (in house)	IFA	1:2
α-rabbit-HRP (Sigma)	Western	1:1000
α-mouse-HRP (Sigma)	Western	1:7000
α-rabbit IRDye 800CW (LiCor)	Western	1:7500
α-mouse IRDye 800CW (LiCor)	Western	1:7500
α-rabbit IRDye 680 (LiCor)	Western	1:7500
α-mouse IRDye 680 (LiCor)	Western	1:7500
α-rabbit Alexa fluor 488 (Life Technologies)	IFA	1:1000
α-mouse Alexa fluor 488 (Life Technologies)	IFA	1:1000
α-rabbit Cy5 (Jackson ImmunoResearch)	FACS	1:1000
α-mouse FITC (Sigma)	FACS	1:1000

### Western blotting

Lysates were prepared by centrifuging cells at 1000g for 5 minutes, washing in PBS and resuspending in Laemmli buffer at 3x10^5^ cells/μl. For PAD1 expression analysis, samples were sonicated using a Bandelin Sonorex Ultrasonic bath to reduce viscosity. All other samples were incubated at 95°C for 5 minutes. Lysates were stored at -20°C until use. Protein was transferred from acrylamide gels to nitrocellulose membrane (GE Healthcare) using a Bio-Rad Mini Trans-Blot cell according to the manufacturer’s instructions, using cold transfer buffer (25 mM Tris, 200 mM glycine, 20% methanol) and run at 200 mA for 90 minutes with stirring.

For reaction, the membrane was blocked at room temperature with agitation for at least 30 minutes in 5% milk in PBS-0.1% Tween 20 (PBS-T). It was then incubated in primary antibody in 5% milk in PBS-T for minimum 1 hour with agitation and washed 3 times in PBS-T.

Secondary detection of the antibody used one of two systems. Either the membrane was incubated in secondary antibody conjugated to horseradish peroxidase (HRP) diluted in 5% milk in PBS-T for 1 hour with agitation and washed three times with PBS-T; the membrane was then reacted with Pierce ECL2 Western Blotting substrate for 2 minutes and exposed to X-ray film (Fujifilm). Alternatively, the signal was visualised using the LI-COR Odyssey system. In this case, the secondary antibody was conjugated to a fluorescent dye and diluted in 50% Odyssey Blocking Buffer/50% PBS-T. After 1 hour incubation in secondary antibody, the membrane was washed twice in PBS-T and once in PBS, and scanned using a LI-COR Odyssey imager.

### Northern blotting

For Northern blotting, RNA was extracted from *Trypanosoma brucei* using the QIAGEN RNeasy kit according to the manufacturer’s instructions and run on a formaldehyde gel in MOPs buffer.

RNA was then transferred from the gel to a positively charged nylon membrane and cross-linked using a UV Stratalinker. The blot was then pre-hybridised in 10 ml formaldehyde hybridisation buffer (5XSSC, 50% formamide, 0.02% SDS, 2% DIG block) at 68°C for 1 hour in a hybridisation oven (Techne Hybridiser HB-1D). This was then replaced with 7 ml hybridization buffer containing 1 μl DIG labelled riboprobe (Roche; prepared according to the manufacturer’s instructions), which had been denatured for 5 mins at 95°C, and hybridised overnight.

The blot was washed at 68°C in the hybridisation tube, first in 2x SSC/0.1% SDS three times for 30 minutes and then once in 0.5x SSC/0.1% SDS for 30 minutes. It was then removed from the tube and rinsed in maleic acid buffer with 0.3% Tween 20 for 1 minute at room temperature. Signal was detected according to the Roche DIG-labelling protocol.

### Phosphoproteomic analysis

For preparation of phosphoproteomic samples, low binding tips and low binding eppendorfs were used. Samples were extracted from 2 replicates each of *T*. *brucei* EATRO1125 AnTat 1.1 90:13 or MEKK1 KO lines. For each, 2.7x10^8^ cells at 9x10^5^/ml were spun at 1000 g for 10 minutes at 4°C, washed three times in PBS and resuspended in 100 μl lysis buffer (4% SDS; 25 mM Tris(2-carboxyethyl)phosphine (TCEP) (Thermo); 50 mM N-ethylmaleimide (Thermo); 150 mM NaCl;1x PhosSTOP phosphatase inhibitor (Roche); 10 mM Na_2_HPO_4_ pH6). The samples were sonicated using a Bioruptor (Diagenode; 10 cycles of 30 seconds alternating sonication/rest at 18°C).

The samples were then chloroform:methanol precipitated. After 10 minutes incubation at 65°C, 400 μl methanol was added and the samples vortexed. 100 μl of chloroform was added and the samples vortexed again. 300 μl of ddH_2_O was added and the samples vortexed for 1 minute. They were then centrifuged for 5 minutes at 9000g and the upper phase aspirated. A further 300 μl of methanol was added and the samples vortexed and centrifuged as before. The supernatants were aspirated and the pellets air dried.

For tryptic digestion, the pellets were resuspended in 150 μl 8M urea/0.1 M Tris pH8/1 mM CaCl_2_ and quantified by Bradford assay. The protein concentration was determined by comparison to a bovine serum albumin (BSA) standard curve (0.1–2.5 mg/ml). 125 μl of Bradford Reagent (Sigma) was added to 2.5 μl protein sample in triplicate in a 96 well plate and absorbance read at 595 nm using a Fluostar Omega plate reader (BMG Labtech). 800 μg of protein for each sample was then diluted with 0.1 M Tris pH8/1 mM CaCl_2_ to 1 M urea and 8 μl LysC (Wako) added. The samples were digested overnight at 37°C. 8 μg trypsin (Pierce) in 50 mM acetic acid was then added and the samples incubated for 4 hours at 37°C. Finally, 1% v/v trifluoroacetic acid was added.

Labelling and mass spectrometry were carried out at the FingerPrints Proteomics Facility at the University of Dundee. The samples purified by solid phase extraction and then quantified by bicinchoninic acid assay prior to labeling with isobaric tandem mass tags (6-plex TMT). The samples were pooled and fractionated into fractions using hydrophilic interaction liquid chromatography.

5% of each fraction was analysed by nLC-MS/MS (nano liquid chromatography-tandem mass spectrometry) using a Thermo Q-Exactive HF mass spectrometer to generate proteomic quantitation. Phosphopeptides were enriched in the remaining 95% of each fraction and these samples were then also analysed by nLC-MS/MS to generate quantitation of phosphopeptides.

### Bioinformatic analysis

Statistical analysis of the fold change in phosphorylation between *MEKK1* KO and parental replicates was performed using the R package limma [59, 60]. This uses the empirical Bayes method which calculates a moderated t-statistic. In this method, posteriors residual SDs replace ordinary SDs, effectively compressing the variance of the peptides with similar means towards a common value, allowing a more stable statistical interference when only few measurement are available [61]. Prior the statistical analysis, data were normalised using the voom methodology included in the limma library. All phosphoproteins that showed an adjusted p-value < 0.05 and a greater than 1.5 fold change compared to parental *T*. *brucei* replicates were considered to be differentially regulated.

### Statistical analysis

Statistical analysis for all parasitaemia, cell cycle, PAD1 IFA, FACS data collected in triplicate was carried out using either Minitab version 15 or GraphPad Prism version 6. Where only a single timepoint was analysed, the data was analysed by students t test. In the case of multiple timepoints, the data was analysed by general linear model with Tukey’s test for multiple comparisons. P values of less than 0.05 were considered statistically significant.

## Supporting information

S1 Table Changes in the phosphoproteome in *MEKK1* null mutants with respect to the *T*. *brucei* EATRO 1125 AnTat1.1 90:13 parent. (XLSX)

S1 Fig Validation of the null mutants used in this study. **A.** The upper schematic shows the strategy for deletion of the two tandemly arranged *RBP7* genes via nested integrated insertion of drug resistance cassettes. For the lower panel, DNA was extracted from *T*. *brucei* EATRO 1125 AnTat1.1 90:13 parasites and amplified using primers detecting either a control gene *TbHYP2* (Tb927.9.4080) (upper panel; detecting an amplicon of 430bp) or *RBP7* (lower panel; detecting an amplicon of 300bp). Two distinct null mutant clones (A, B) are shown. B. A southern blot of genomic DNA from *T*. *brucei* EATRO 1125 AnTat1.1 90:13 wild type cells, *YAK* single allele knockout (sKO) and *YAK* double allele knock out (dKO) cells hybridised with a *YAK*-specific DNA probe. C. PCR based assay validating creation of a null mutant for the *MEKK1* cell line. DNA was extracted from *T*. *brucei* EATRO 1125 AnTat1.1 90:13 parasites and amplified using primers detecting either *MEKK1* (upper panel; detecting an amplicon of 100bp) or a control gene *TbHYP2* (Tb927.9.4080) (lower panel; detecting an amplicon of 430bp).
(TIF)

S2 Fig Ectopic expression of RBP7B reduces growth in RBP7AB and YAK null mutants *in vitro*. Null mutants for *RBP7AB* (Panel A) and *YAK* (Panel B) were grown *in vitro*, with the ability of RBP7B inducible ectopic expression to slow growth of the null mutant lines assayed in each case. The relative expression of RBP7B in the *RBP7AB* null or *YAK* null mutant is shown in Panel C.
(TIF)

S3 Fig Growth profiles upon PP1 OE in different cell lines. A. Growth *in vitro* of parental *T*. *brucei* EATRO 1125 AnTat1.1 90:13 cells induced (+tet; dashed lines) or not (-tet; solid lines) to express PP1-6. B. Growth *in vitro* of *YAK* null mutant cells induced (+tet; dashed lines) or not (-tet’ solid lines) to express PP1-6.
(TIF)

S4 Fig % 1K1N cells in each of the cell lines induced (dox+) or not (dox-) to express PP1-6 in the parental wild type *T*. *brucei* EATRO 1125 AnTat1.1 90:13 cells (A), or the *RBP7AB* (B) or *YAK* (C) null mutant lines. Data represent analyses of triplicate infections, and are derived from the same infections shown in Figs 4 and 5.
(TIF)

S5 Fig Growth profiles upon NEK OE in different cell lines. Growth profiles of cells with inducible ectopic expression of NEK17 in parental *T*. *brucei* EATRO 1125 AnTat1.1 90:13 cells (Panel A), *RBP7AB* null mutants (Panel B) or *YAK* null mutants (Panel C). Panel D shows the expression of NEK17 detected with BB2 antibody recognising the Ty1 epitope tag incorporated into NEK17 when NEK17 is induced (tet+) for expression in parental cells (NEKOE), RBP7 null mutants (RBP7KO NEK OE) or YAK null mutants (YAK KO NEK OE). EF1 alpha provides the loading control.
(TIF)

S6 Fig TbTOR4 depletion causes slowed growth and stumpy formation in pleomorphic *T*. *bru*c*ei*. A. RNAi targeting *TbTOR4* causes slowed growth and premature stumpy formation in *T*. *brucei* EATRO 1125 AnTat1.1 90:13 cells. *TbTOR4* RNAi induced *(*◼), *TbTOR4* RNAi uninduced (●). A schematic representation of the morphology of the parasites at day 6 post infection is shown. B. Northern blot of the expression levels of *TbTOR4* on parasites harvested on day 6 of infection from mice where RNAi was induced (+dox) or not induced (-dox). The rRNA of the respective samples is shown also as a loading control.
(TIF)

S7 Fig NEK17 is differentially phosphorylated between parental cells and MEKK null mutants. The sequence of *NEK17* is shown annotated by key domains associated with protein kinase function, which are highlighted and colour coded according to the Table below the sequence. The differentially phosphorylated Threonine 195 residue is highlighted in red and underlined.
(TIF)
