# Supplementary figures and images for: Non-linear hierarchy of the quorum sensing signalling pathway in bloodstream form African trypanosomes
Source: PLoS Pathog. 2018 Jun 25;14(6):e1007145. doi: 10.1371/journal.ppat.1007145 (PMC6034907; doi:10.1371/journal.ppat.1007145)

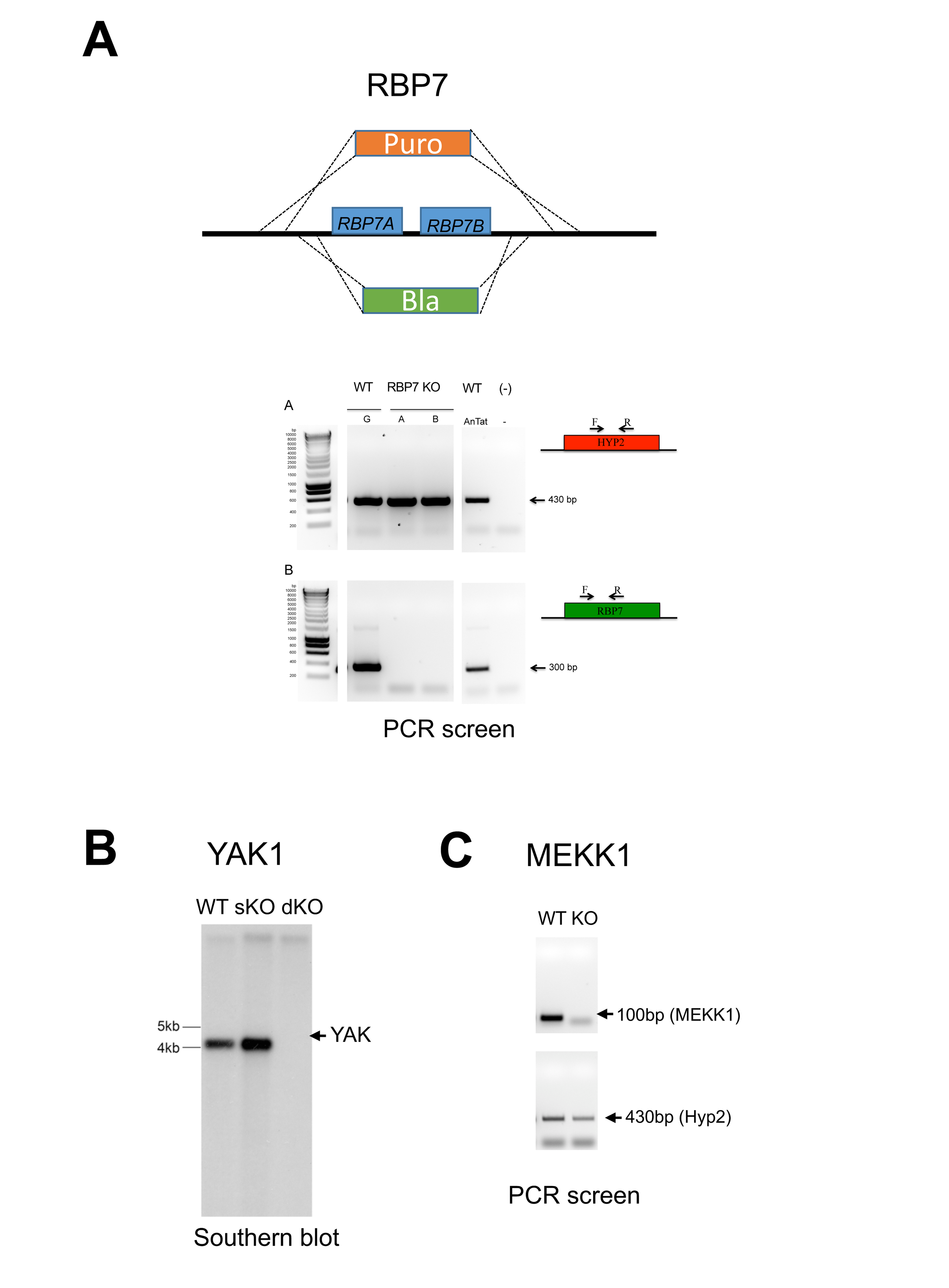

Supplement: S1 Fig — A. The upper schematic shows the strategy for deletion of the two tandemly arranged RBP7 genes via nested integrated insertion of drug resistance cassettes. For the lower panel, DNA was extracted from T. brucei EATRO 1125 AnTat1.1 90:13 parasites and amplified using primers detecting either a control gene TbHYP2 (Tb927.9.4080) (upper panel; detecting an amplicon of 430bp) or RBP7 (lower panel; detecting an amplicon of 300bp). Two distinct null mutant clones (A, B) are shown. B. A southern blot of genomic DNA from T. brucei EATRO 1125 AnTat1.1 90:13 wild type cells, YAK single allele knockout (sKO) and YAK double allele knock out (dKO) cells hybridised with a YAK-specific DNA probe. C. PCR based assay validating creation of a null mutant for the MEKK1 cell line. DNA was extracted from T. brucei EATRO 1125 AnTat1.1 90:13 parasites and amplified using primers detecting either MEKK1 (upper panel; detecting an amplicon of 100bp) or a control gene TbHYP2 (Tb927.9.4080) (lower panel; detecting an amplicon of 430bp). (TIF) [file ppat.1007145.s002.tif]

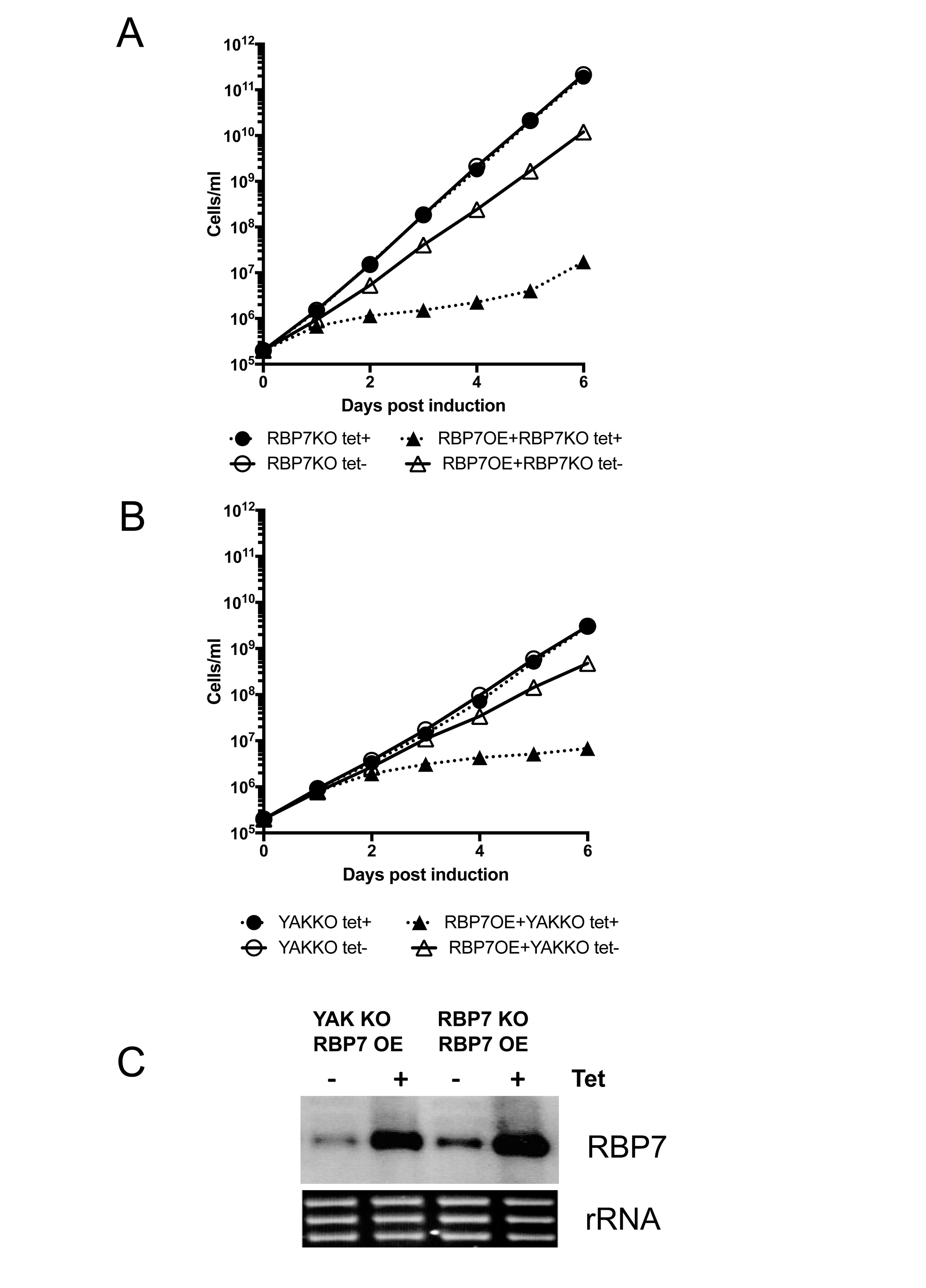

Supplement: S2 Fig — Null mutants for RBP7AB (Panel A) and YAK (Panel B) were grown in vitro, with the ability of RBP7B inducible ectopic expression to slow growth of the null mutant lines assayed in each case. The relative expression of RBP7B in the RBP7AB null or YAK null mutant is shown in Panel C. (TIF) [file ppat.1007145.s003.tif]

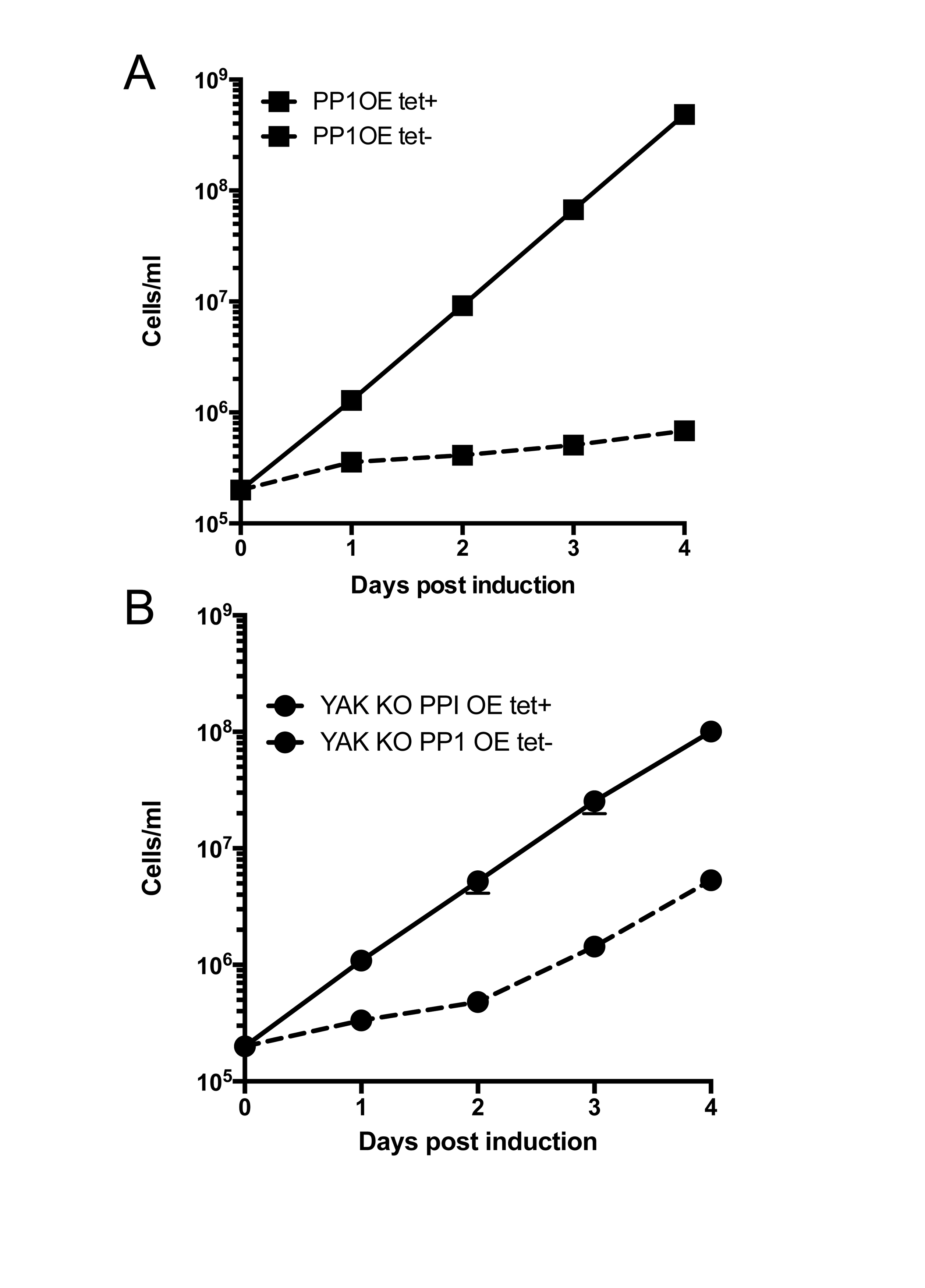

Supplement: S3 Fig — A. Growth in vitro of parental T. brucei EATRO 1125 AnTat1.1 90:13 cells induced (+tet; dashed lines) or not (-tet; solid lines) to express PP1-6. B. Growth in vitro of YAK null mutant cells induced (+tet; dashed lines) or not (-tet’ solid lines) to express PP1-6. (TIF) [file ppat.1007145.s004.tif]

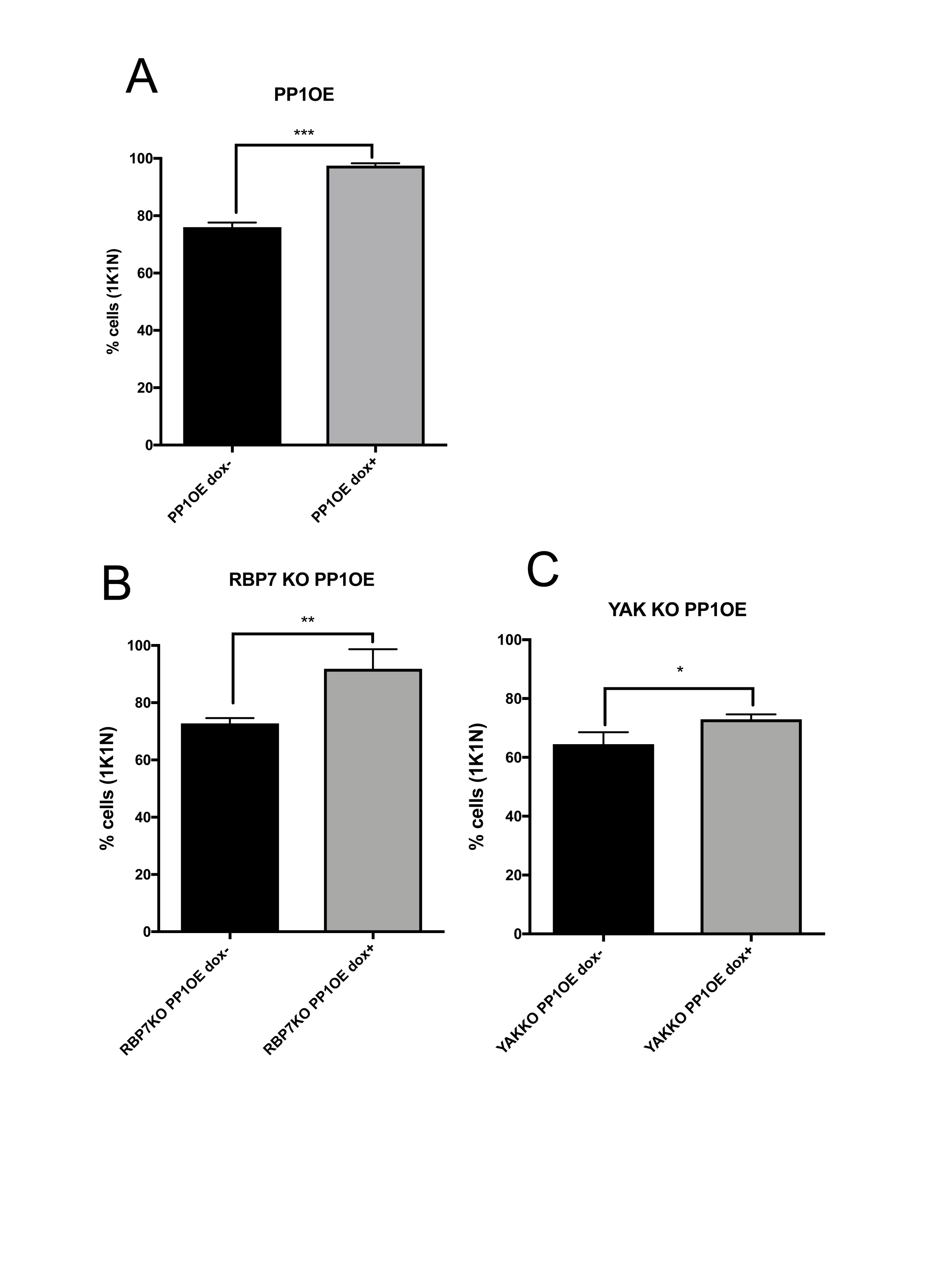

Supplement: S4 Fig — % 1K1N cells in each of the cell lines induced (dox+) or not (dox-) to express PP1-6 in the parental wild type T. brucei EATRO 1125 AnTat1.1 90:13 cells (A), or the RBP7AB (B) or YAK (C) null mutant lines. Data represent analyses of triplicate infections, and are derived from the same infections shown in Figs 4 and 5. (TIF) [file ppat.1007145.s005.tif]

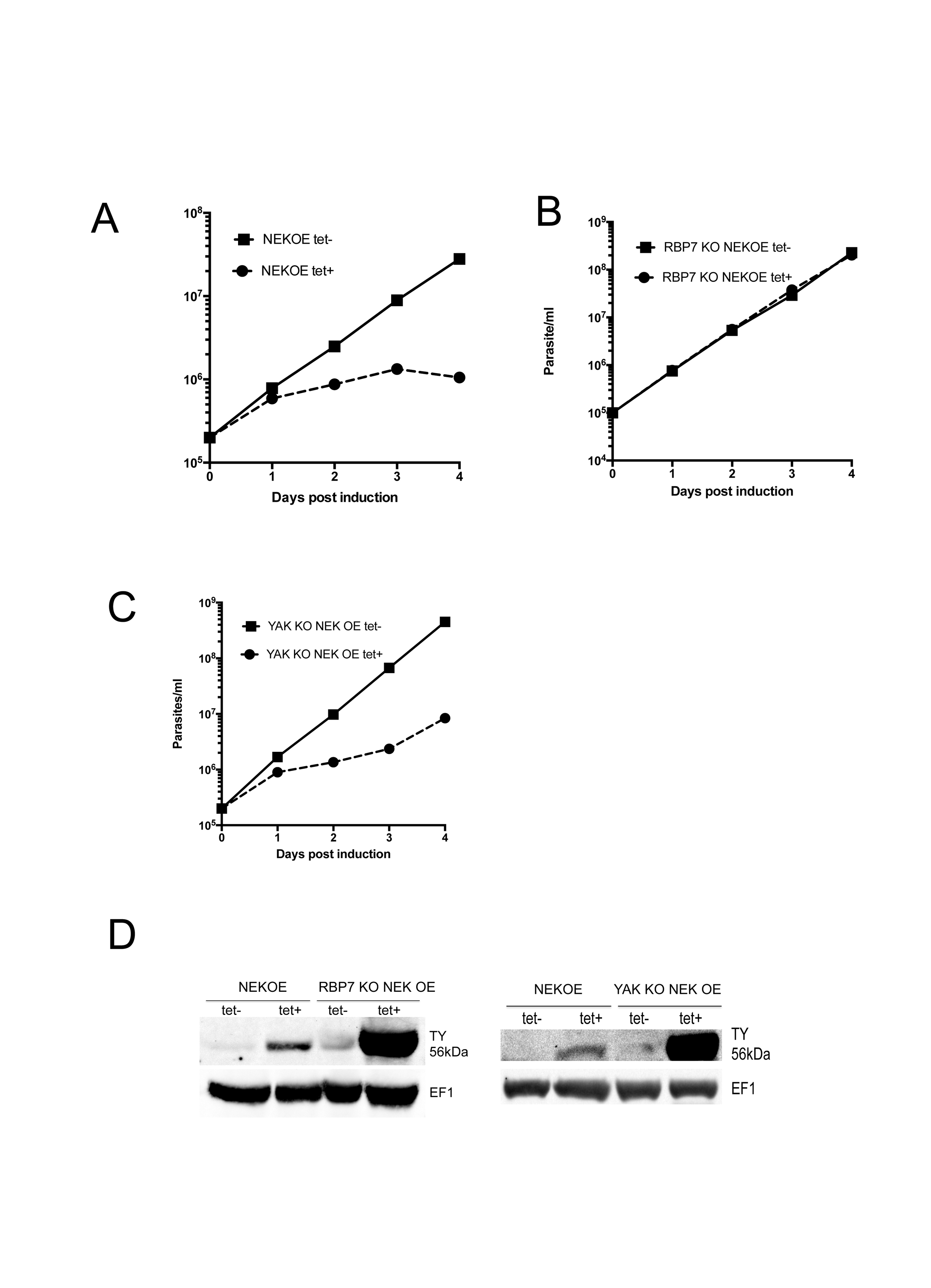

Supplement: S5 Fig — Growth profiles of cells with inducible ectopic expression of NEK17 in parental T. brucei EATRO 1125 AnTat1.1 90:13 cells (Panel A), RBP7AB null mutants (Panel B) or YAK null mutants (Panel C). Panel D shows the expression of NEK17 detected with BB2 antibody recognising the Ty1 epitope tag incorporated into NEK17 when NEK17 is induced (tet+) for expression in parental cells (NEKOE), RBP7 null mutants (RBP7KO NEK OE) or YAK null mutants (YAK KO NEK OE). EF1 alpha provides the loading control. (TIF) [file ppat.1007145.s006.tif]

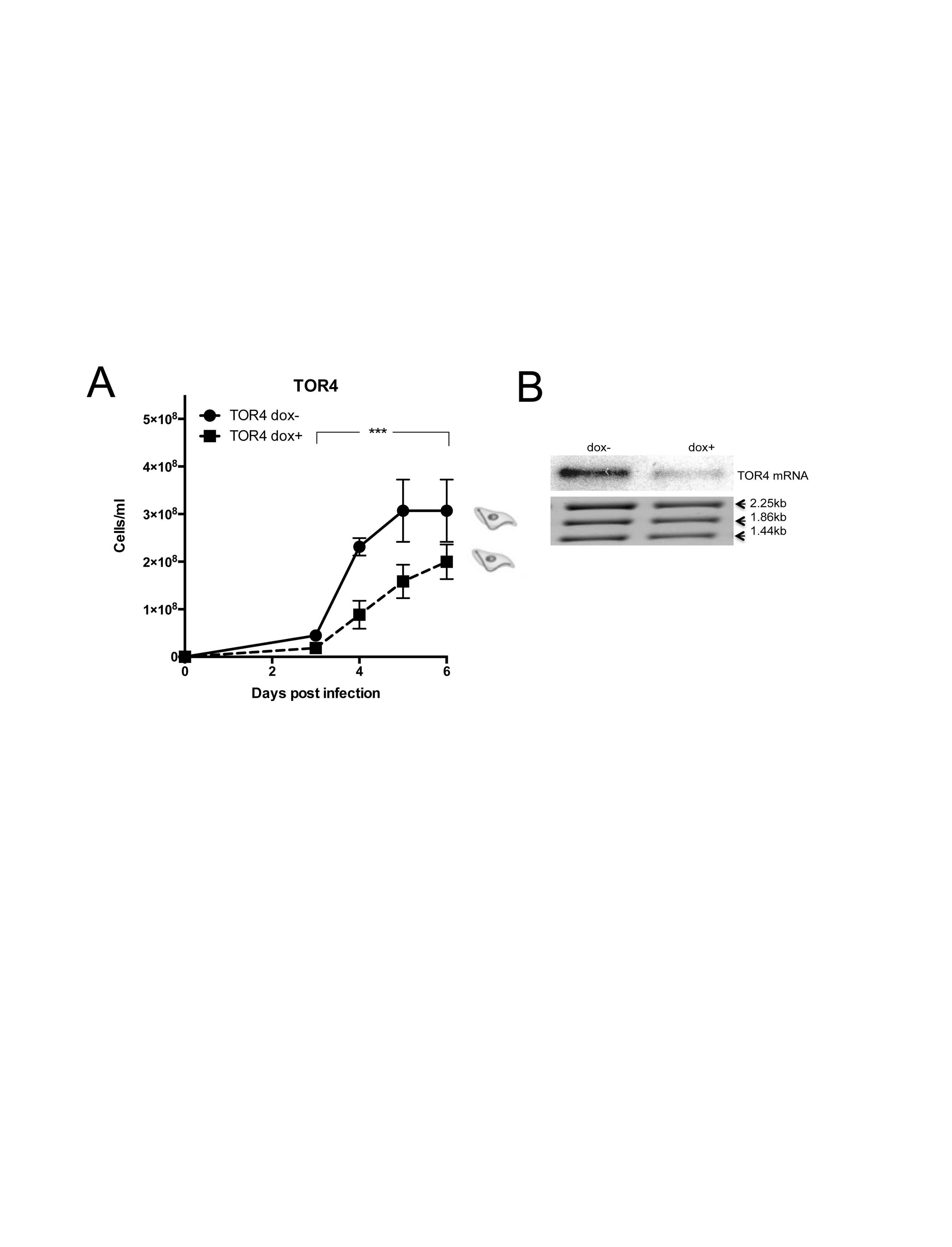

Supplement: S6 Fig — A. RNAi targeting TbTOR4 causes slowed growth and premature stumpy formation in T. brucei EATRO 1125 AnTat1.1 90:13 cells. TbTOR4 RNAi induced (◼), TbTOR4 RNAi uninduced (●). A schematic representation of the morphology of the parasites at day 6 post infection is shown. B. Northern blot of the expression levels of TbTOR4 on parasites harvested on day 6 of infection from mice where RNAi was induced (+dox) or not induced (-dox). The rRNA of the respective samples is shown also as a loading control. (TIF) [file ppat.1007145.s007.tif]

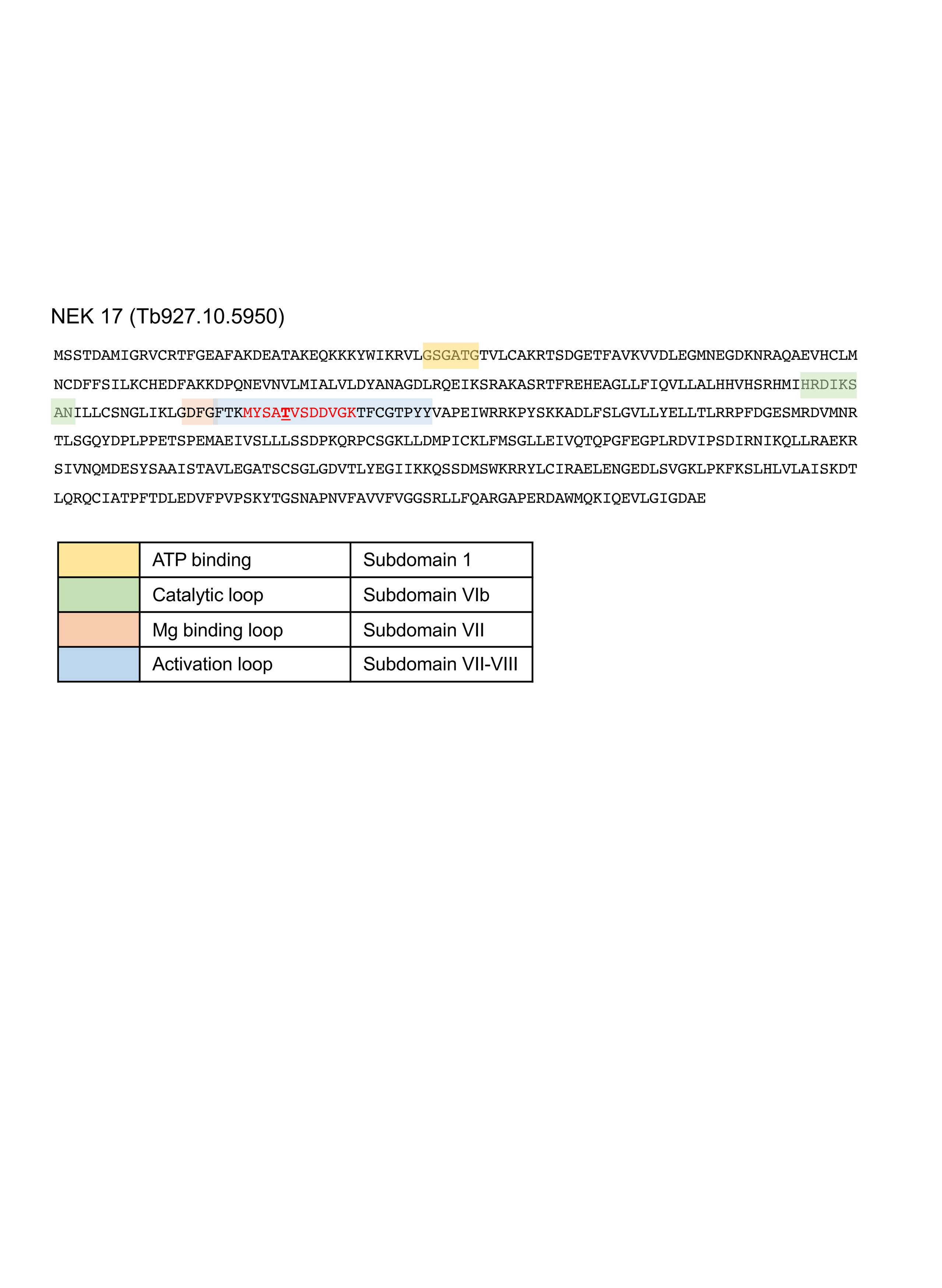

Supplement: S7 Fig — The sequence of NEK17 is shown annotated by key domains associated with protein kinase function, which are highlighted and colour coded according to the Table below the sequence. The differentially phosphorylated Threonine 195 residue is highlighted in red and underlined. (TIF) [file ppat.1007145.s008.tif]
